# Diet of a threatened endemic fox reveals variation in sandy beach resource use on California Channel Islands

**DOI:** 10.1371/journal.pone.0258919

**Published:** 2021-10-28

**Authors:** Henry M. Page, Juliann Schamel, Kyle A. Emery, Nicholas K. Schooler, Jenifer E. Dugan, Angela Guglielmino, Donna M. Schroeder, Linnea Palmstrom, David M. Hubbard, Robert J. Miller

**Affiliations:** 1 Marine Science Institute, University of California, Santa Barbara, California, United States of America; 2 Channel Islands National Park, Ventura, California, United States of America; 3 Department of Ecology and Evolutionary Biology, University of California, Los Angeles, California, United States of America; 4 Bureau of Ocean Energy Management, Camarillo, California, United States of America; Hawaii Pacific University, UNITED STATES

## Abstract

The coastal zone provides foraging opportunities for insular populations of terrestrial mammals, allowing for expanded habitat use, increased dietary breadth, and locally higher population densities. We examined the use of sandy beach resources by the threatened island fox (*Urocyon littoralis*) on the California Channel Islands using scat analysis, surveys of potential prey, beach habitat attributes, and stable isotope analysis. Consumption of beach invertebrates, primarily intertidal talitrid amphipods (*Megalorchestia* spp.) by island fox varied with abundance of these prey across sites. Distance-based linear modeling revealed that abundance of giant kelp (*Macrocystis pyrifera*) wrack, rather than beach physical attributes, explained the largest amount of variation in talitrid amphipod abundance and biomass across beaches. δ^13^C and δ^15^N values of fox whisker (vibrissae) segments suggested individualism in diet, with generally low δ^13^C and δ^15^N values of some foxes consistent with specializing on primarily terrestrial foods, contrasting with the higher isotope values of other individuals that suggested a sustained use of sandy beach resources, the importance of which varied over time. Abundant allochthonous marine resources on beaches, including inputs of giant kelp, may expand habitat use and diet breadth of the island fox, increasing population resilience during declines in terrestrial resources associated with climate variability and long-term climate change.

## Introduction

Insular populations of terrestrial mammals are often subject to deleterious effects of fluctuations in food supply, pathogens, non-native species, and habitat modifications that can dramatically affect their dynamics and long-term viability [1–4]. For these mammals, the coastal zone may provide beneficial foraging opportunities that increase dietary breadth, mitigating some of the environmental challenges of island life and allowing for locally higher population densities [5–7]. Sandy beaches are ecotonal environments that lack appreciable *in situ* primary production with endemic invertebrate populations subsidized by inputs of organic matter from marine ecosystems [8,9]. The exploitation of these intertidal and supralittoral invertebrates, and whether their use is occasional and opportunistic or more sustained remains understudied.

**Fig 1:**
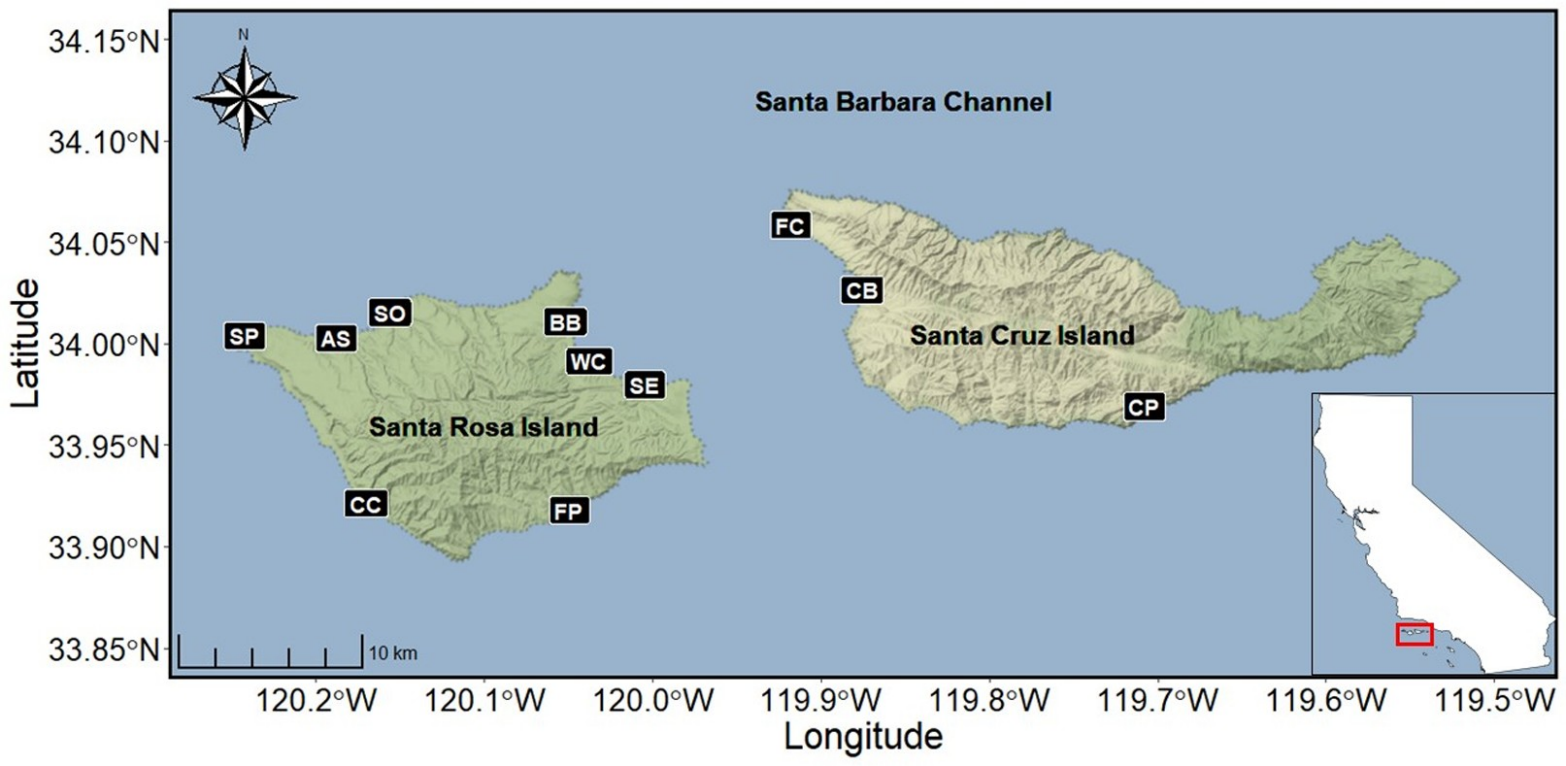
Locations of the study beaches on Santa Rosa and Santa Cruz Islands off the California, USA, coastline (inset). Abbreviations: SP—Sandy Point, SO—Soledad, AS—Arlington Springs, BB—Bechers Bay, WC—Water Canyon, SE—Southeast Anchorage, FP—Ford Point, CC—China Camp, FC—Forney’s Cove, CB—Christy Beach, CP—Coches Prietos. Attribution: Map tiles by Stamen Design, under CC BY 3.0. Data by OpenStreetMap, under OdbL.

The endemic island fox (*Urocyon littoralis*), a diminutive descendant (approximately 50–70% the weight) of the mainland gray fox (*U*. *cinereoargenteus*) [10,11], is a generalist predator that inhabits, as six different subspecies, six of the eight California Channel Islands in the Southern California Bight (Santa Catalina, San Clemente, Santa Cruz, San Miguel, San Nicolas, and Santa Rosa) [12]. The subspecies of island fox on the northern islands of Santa Cruz, Santa Rosa, and San Miguel (Fig 1) were listed as federally endangered in 2004, following precipitous declines in population sizes due to predation by golden eagles (*Aquila chrysaetos*), facilitated by the presence of non-native ungulates [2,13]. Management actions to prevent the extirpation of the island fox targeted the removal of the ungulates and golden eagles, the re-introduction of bald eagles (*Haliaeetus leucocephalus*), the chief competitor of golden eagles, and an *in situ* captive breeding program for foxes that enabled fox populations to increase such that these subspecies were de-listed as Endangered Species in September 2016 [14]. Currently, the island fox is listed as “near threatened” on the ICUN Red List of Threatened Species [15] and “threatened” by the State of California and is protected by California State Law [16].

Typical of other fox species, island fox are considered generalist consumers with a diet consisting largely of terrestrial foods, including fruits, insects, and deer mice [11,17–19]. However, the importance of marine resources to fox diet is largely unknown. Cypher et al. [19] found trace amounts of pinniped remains in island fox scat from five islands, and crustacean remains (crabs, amphipods) were present in some fox scats from San Clemente and Santa Rosa Islands [18,19], indicating that foxes foraged along the shoreline. The possible use of marine carrion, acquired either from association with ancient humans, or through foraging along the shore, is cited as a likely marine food source for island fox in archaeological studies [20,21]. Yet, little data exist to date that suggest appreciable use of marine sources by ancient [21] or contemporary island fox [11,18,19]. The reported importance of marine subsidies, which appear to be rare or occasional to fox nutrition [19], may depend on context as the contemporary dietary studies of island foxes have focused within the larger terrestrial landscape.

The exploitation of beach resources by terrestrial mammals, including island fox, may be associated with local beach productivity and conditions affecting the composition, abundance, and accessibility of potential food resources. Marine carrion represents a direct subsidy to foxes, in the form of species that generally feed offshore (e.g. pinnipeds, fish, subtidal invertebrates, seabirds). However, intertidal beach invertebrates have received scant consideration as potential food sources despite the often high abundance and biomass of these potential prey [8,22,23]. These include suspension feeding sand crabs and bivalves sustained by allochthonous inputs of plankton [8,22], and upper intertidal invertebrates reliant on kelp and other macrophyte detritus originating from subtidal rocky reefs.

Coastal landscape heterogeneity may also influence the availability and exploitation of beach food resources by island fox and other terrestrial mammals. For example, Kimber et al. [24] reported that red foxes (*Vulpes vulpes*) were more likely to be found on ocean beaches characterized by high dunes and large expanses of vegetation. Schlacher et al. [25] reported the beach-dune interface as a hotspot of foraging activity by a variety of terrestrially-based scavengers, including red fox, with less foraging activity in adjoining dunes. Landscape and marine features that influence the delivery and retention of macroalgal wrack also have the potential to drive the availability of beach resources to terrestrial predators. Beds of giant kelp (*Macrocystis pyrifera*), which supply the majority of macroalgal wrack to beaches in the region [8,26] are spatially variable [27–29] and beach morphology and orientation relative to prevailing currents and wind may strongly influence the delivery and retention of kelp wrack on the beach [30,31].

Although island fox home ranges can border the shoreline [32–34], previous studies have not explicitly assessed fox diet in the shoreline-upland ecotone to evaluate the relationship between marine food resources available to island fox, fox use of these resources, and the influence of landscape features. Since island fox home ranges are small, typically < 1 km^2^ ([32,33], but see [34]) and can border the coast, beach resources could play an important role in the diet of coastal foxes, influencing diet breadth and performance metrics, as has been suggested for coyotes (*Canis latrans*) foraging along the shoreline of Año Nuevo, northern California [7].

In this study, we explored the following hypotheses using sandy beach study sites on Santa Rosa and Santa Cruz Islands: 1) endemic upper beach invertebrates are exploited by island fox, with use related to the abundance and biomass of these taxa, 2) the availability of upper beach invertebrates, and therefore their use as food, varies with biological and physical elements of the landscape, and 3) beach prey resources, in general, can comprise a stable contribution to island fox diet, and increase individual and population level isotopic niche width, a proxy for diet breadth, which may affect fox body weight and condition.

## Materials and methods

### Study area and sites

The California Channel Islands, USA, form a unique archipelago in the Southern California Bight [3,35]. This study was conducted on Santa Rosa and Santa Cruz Islands, two of the four northern Channel Islands (Fig 1). Santa Rosa Island (area = 217 km^2^) and Santa Cruz Island (249 km^2^), located approximately 9 km to the east, are topographically complex. The hilly and mountainous terrain of both islands is incised by deep ravines and surrounded by shorelines comprised of sandy beaches, rocky intertidal benches, and steep cliff faces with vertical intertidal habitat. Approximately 33% of the shoreline of Santa Rosa Island and 14% of shoreline on Santa Cruz Island is sandy beach, as measured using the 1981 USGS 7.5 minute orthophotoquads and topographic maps of these islands. Broad sandy beaches are present on the northwest, northeast and southwest shores of Santa Rosa Island, whereas longer stretches of sandy beach are restricted to the western and southern shores of Santa Cruz Island.

The northern Channel Islands experience a Mediterranean climate with dry, foggy summers, and cool, rainy winters [35,36]. Average rainfall is approximately 38 cm on Santa Rosa and 51 cm on Santa Cruz Island, but rainfall is highly variable with years of extreme drought contrasting with years of exceptionally high rainfall. Both islands are home to numerous endemic plant and animal species, including the island fox. Further descriptions of the physical and biological characteristics of Santa Rosa and Santa Cruz Islands are provided by [36] and [35].

Our study included seven beaches on Santa Rosa Island and three beaches on Santa Cruz Island (Table 1 and Fig 1). The sites on Santa Rosa Island encircled the island and varied in size and morphology from relatively short, narrow and bluff-backed beaches (e.g., Southeast Anchorage, Water Canyon, Ford Point) to longer, wider beaches backed by dune fields, bluffs or both (e.g., Soledad, Sandy Point, China Camp) (Table 1). The sites on Santa Cruz Island included an expansive, exposed dune-backed beach at the west end of the island (Christy Beach), a protected cove (Forney’s Cove), and a smaller, protected south-facing pocket beach at Coches Prietos (Table 1 and Fig 1).

**Table 1:** Locations and values for physical and biological characteristics of sandy beach study sites on Santa Rosa and Santa Cruz Islands, California, USA.

			Beach characteristics				
Site	Lat, Long	*Macrocystis wrack (m* ^*-2*^ *m* ^*-1*^ *)*	Offshore kelp canopy (kg)	Beach length (km)	Orientation (°)	Upper beach width (m)	HTS slope (°)
Santa Rosa Island							
Sandy Point	34.006, -120.240	3.57 ± 0.97	37786.3	1.15	322	18.1 ± 0.67	0.6 ± 1.11
Soledad	34.011, -120.161	2.17 ± 0.34	725183.1	1.93	324	21.93 ± 0.48	-0.53 ± 0.77
China Camp	33.921, -120.170	1.64 ± 0.29	619559.4	1.25	232	26.83 ± 2.22	1.93 ± 1.17
Ford Point	33.918, -120.049	0.11 ± 0.05	0.0	0.26	117	4.13 ± 0.79	6.9 ± 4.85
Bechers Bay	34.008, -120.048	0.11 ± 0.05	41535.5	0.36	56	6.50 ± 3.77	3.367 ± 1.71
Water Canyon	33.994, -120.041	0.15 ± 0.05	8057.7	2.25	49	13.23 ± 1.64	3.7 ± 1.18
SE Anchorage	33.980, -120.011	0.47 ± 0.08	0.0	0.16	21	3.5	5.0
Santa Cruz Island							
Forney Cove	34.058, -119.919	0.09 ± 0.04	6116.8	0.78	167	17.43 ± 2.43	3.53 ± 0.28
Christy Beach	34.024, -119.877	0.41 ± 0.20	17042.8	1.90	286	13.17 ± 0.17	6.43 ± 0.55
Coches Prietos	33.969, -119.708	0.24 ± 0.09	1250.3	0.26	134	12.23 ± 2.07	4.57 ± 0.75

Mean values ± 1SE. HTS = high tide strand line.

### Site characteristics

Orientation and beach length were measured for each beach site. We measured beach orientation as the compass heading of the shore-normal line (0°/360° = North) in Google Earth Pro (v. 7.3). Beach orientation strongly influences exposure to wind and mean current flow. In the region of our study, coastal waters offshore of north- and northwest-facing beaches experience high prevailing southeast current flows [37]. Beach length, also measured in Google Earth, was defined as the sandy shoreline distance (km) of continuous beach at each site, which was typically bounded by rocky headlands.

We measured physical attributes and quantified the abundance of macrophyte wrack on the beach and beach invertebrates at each study site during daytime low tides in August—September 2018 coincident with the sampling of fox scat (see below). Physical measurements and biological sampling were conducted along five (SRI) or three (SCI) randomly spaced transects run perpendicular to the water line from the landward boundary of the beach to the swash following methods modified from [8]. Physical variables measured on each transect included width of the upper beach zone, and beach slope at the high tide strand line (HTS). Upper beach width, defined as the distance between the landward boundary of the beach and the high tide strandline (HTS), was measured along each transect and calculated as the mean upper beach width for each study beach. Beach slope was measured for each transect at the HTS using a digital level and averaged across transects.

Biomass of giant kelp beds offshore that could potentially supply wrack to the study beaches was estimated using Landsat imagery [27,29,38]. We calculated mean kelp canopy biomass (kg) for 60 m x 60 m patches that represent the aggregation of 1–4 Landsat pixels for all images with usable data (i.e. no clouds) for the three months preceding our sampling (July to September 2018). These data were mapped using ArcMap in ArcGIS (v. 10.8.1). The kelp forest patches directly offshore and/or immediately adjacent to the study beaches were identified and the biomass values summed to provide a total mean kelp canopy biomass for the offshore area surrounding each study site.

### Beach wrack and invertebrates

The abundance of stranded drift kelp *Macrocystis pyrifera*, other macrophytes (e.g., other macroalgae, surfgrass), and other organic (i.e., driftwood) and inorganic (e.g., plastic) material on the beach was estimated using line-intercept along the above transects [8]. Abundance values for the above categories of wrack were calculated using transects as replicates at each site and expressed as mean cover per area (m^2^) per meter wide strip of beach. To estimate the abundance of upper beach, wrack associated, and swash zone invertebrate prey, which tend to occupy distinct zones, we stratified the beach into an upper beach zone, a talitrid amphipod zone, and a sand crab zone that included the swash zone.

Talitrid amphipods of the genus *Megalorchestia* are numerically the most important consumer of drift kelp on the beaches of southern California [8,26] with abundances exceeding 10,000 individuals m^-1^ in some locations [23]. As are many beach invertebrate taxa, talitrid amphipods are generally nocturnal, burrowing into the sand or hiding under wrack during the day and emerging at night [39]. The talitrid amphipod zone extended from the seaward to the landward boundary of the burrows occupied by these amphipods during the day. The upper beach zone was located higher on the beach than the talitrid zone, extending from landward boundary of the talitrid zone to the beach-upland ecotone (e.g. dune vegetation, bluffs, cobble berm) and contained drier wrack and a different assemblage of species than the talitrid zone, including the beach isopods, *Tylos punctatus* and *Alloniscus perconvexus*. Sand crabs (*Emerita analoga*) form aggregations at the low beach and swash zone [8,22]. The sand crab sampling zone extended across the distribution of sand crabs and was not contiguous with the upper beach and talitrid zones.

We sampled beach invertebrate fauna during the day using a 10 cm diameter core pushed into the sediment to a depth of 20 cm. Ten core samples were taken at uniform intervals within each of the talitrid and upper beach zones spaced along each transect to cover the entire zone. The contents of the 10 cores per zone were pooled, sieved through 1.5 mm mesh, returned to the laboratory in freezer bags, and frozen for later processing. After thawing, all macroinvertebrates in the samples were identified to the lowest taxonomic level possible, typically species, enumerated, and weighed to the nearest milligram blotted wet weight. Abundance and wet biomass values were expressed per linear meter of beach as recommended for the highly mobile fauna of this dynamic ecosystem [9].

The abundance and biomass of sand crabs was estimated from twenty uniformly spaced cores (10 cm depth) taken in the swash zone along transects across the distribution of these crabs within two hours of low tide. Core samples from each transect were combined and sieved through 1.5 mm mesh bags. Crabs were enumerated and measured to the nearest 1.0 mm carapace length (CL). Biomass of sand crabs per transect was estimated from the relationship between carapace length and blotted wet weight (wet biomass, g = 0.0003*CL^3^–0.00008*CL^2^ + 0.0004*CL, *r*^2^ = 0.96, n = 306).

We evaluated differences in the abundance and biomass of invertebrate prey taxa among beaches using one-way ANOVA. Data were log (x+1) transformed prior to analysis to satisfy assumptions of homogeneity of variances (*p* > 0.2, Levene’s test). Pairwise differences among beaches were explored using the Šidák *post hoc* test, which adjusts *p* values in multiple pairwise comparisons to reduce type I error [40].

The use of each beach site by live pinnipeds, and the presence and condition (i.e., old vs. fresh) of vertebrate and invertebrate carcasses, was assessed by recording the number and type of carcasses along an up to one-kilometer-long section of each beach (shorter if beach length < 1 km) encompassing the upper and lower intertidal zones. These surveys were conducted in 2018, during our beach and invertebrate sampling, as well as once in August—October of 2016 and 2017.

### Fox scat

To compare the relative contribution of beach and terrestrial foods to the diet of island fox across beach sites, we analyzed island fox scats collected from the 10 beach sites. Scat analysis is commonly used to assess the diet of mammalian consumers, and its drawbacks are well recognized. These include the limitation of dietary inferences to recent meals and foods with indigestible components that can be visually identified and quantified (e.g., bones, hair, exoskeletons) [41,42]. Nevertheless, this approach can be invaluable in describing items ingested by mammalian consumers [11,19,42,43].

We searched for and collected fox scat in August—September 2018 on the upper sandy beach, within the sandy beach-upland ecotone (e.g., dunes), and adjacent upland, including drainage gullies and bluffs, by walking parallel to the shoreline along the upper beach, then backtracking the same distance along parallel routes approximately 10–20 m inland. Tracks were recorded with a handheld GPS device. This was followed by searching selected gullies or dry streambeds at right angles to these transects as well as along accessible bluffs. The total length of the search area varied among sites, ranging from 0.8 to 3.8 km. In general, all scat encountered were collected. Exceptions occurred at locations where several scats were present (fox latrines). There was no way of knowing in our study if the scats collected at spatially separated locations were from different foxes. On collection, GPS coordinates of each scat were recorded, and the scat was transferred to a small plastic bag, labelled and stored at -20°C on return to the laboratory.

Dietary items in the scats were determined following methods modified from [18] and [43]. In the laboratory, each scat was transferred to a cloth bag (mesh size = 0.5 mm), which was soaked in a container filled with warm fresh water for 30 minutes. The cloth bag was then moved to another container filled with fresh water and manually agitated until there was no scat matrix coming out of the bag. The bag and scat were then dried to a constant weight at 60°C. The contents in the bag were emptied into a petri dish, sorted, and identified to the lowest taxonomic level possible under a dissecting microscope with the aid of reference collections and established guides [35,44–46]. Contents were grouped into either terrestrial or beach materials and then weighed. Three arthropod groups endemic to the beach, and recognized from whole bodies or heads were counted: talitrid amphipods *Megalorchestia* spp., isopods *Alloniscus perconvexus* and *Tylos punctatus*, and the staphylinid beetle *Thinopinus pictus*.

We compared the contribution of beach and terrestrial resources to fox diet across sites using the proportion of collected scats containing foods of beach origin at each site and the mean proportion by dry weight of beach versus terrestrial foods in individual scats. The proportion of collected scats containing beach foods was computed for each site by dividing the number of scats with beach foods by the total number of scats collected at each site. The relative contribution of beach versus terrestrial foods in individual scats at each beach was computed by dividing the dry weight of beach foods in each scat by the total weight of the food items in the scat.

### Statistical analyses

We compared the proportion of fox scats containing beach prey items across sites using the Fisher’s Exact Test [47] and the mean proportion of beach prey in individual scats (as dry weight) using nonparametric Kruskal Wallis tests. We compared the percent of scats with beach material and the percent beach material per scat to the abundance and biomass of upper beach invertebrates using weighted linear regression with and without logit transformation of percent values [48]. The logit transformation had minimal effect on the analysis and these results are provided in the supplementary material (S1 Table).

We used distance-based linear models (DistLM) to explore relationships between environmental predictor variables and the abundance and biomass of beach endemic invertebrate prey available to foxes [49]. DistLM is a non-parametric permutational routine that permits an analysis of relationships between species data (response variables) and numerical and categorical predictor variables. DistLM analysis was conducted on a dissimilarity matrix of species data based on Euclidean distances using a step-wise selection procedure, 9999 permutations, and the adjusted R^2^ as the selection criterion [49].

The abundances of the main beach prey found in fox scats—amphipods *Megalorchestia* spp. and isopods *Tylos punctatus* and *Allonoscus periconvexus*, and beetle *Thinopinus pictus* were the response variables in our analysis. These taxa were analyzed combined and separated into two groups, *Megalorchestia* spp. and their beetle predator, *Thinopinus pictus* [50] in one group, and the isopod taxa combined in a second group. Predictor variables included in the DistLM model were posited to either directly or indirectly influence the abundance of invertebrate prey on the beach (and consequently prey available to foxes). Physical variables included beach compass orientation (i.e., orientation to prevailing wind and swell), beach length, width of the upper beach, and slope measured at the high tide strand line (HTS). We transformed beach orientation for use as a predictor variable in our models by taking the cosine and the sine of the compass direction in radians [51]. The sine and cosine were taken as paired terms and used as two predictor variables in the models; cosine terms can be considered as explaining effects operating north to south and sine terms as east to west [52].

Biological variables included the cover of *Macrocystis* wrack and the size (biomass/km^2^) of kelp beds offshore during a three-month window that encompassed the sampling period (July—September 2018). We also included one categorical predictor variable in the analysis, the presence (or absence) of dunes, as defined by the presence of a foredune and/or vegetated hummocks backing the beach. Statisticial analyses were conducted using IBM SPSS 23, the statistical package in R [53], and Permanova+ for Primer [49].

### Fox whisker samples for isotope analysis

To determine if beach foods represent a measurable contribution to island fox nutrition over time, we supplemented the scat analysis with stable isotope analysis of fox whisker segments from two of the sites (Soledad, China Camp) and a third site contiguous with Soledad (Arlington Springs, Fig 1). The dietary history of island fox can be gleaned from stable isotope analysis of their whiskers (vibrissae), which incorporate carbon and nitrogen atoms along their length as they grow, and remain inert once the tissue is built. Longitudinal sampling of whisker segments, similar to hair, captures variability in diet over time and is a more accurate representation of isotopic niche width (see below) than the whole tissue [54]. Isotopic analysis of keratinous tissue segments, such as whiskers and hair, has provided insights into temporal dietary patterns of a variety of mammalian species, including sea otters, red and kit foxes, grizzly bears, and elephants [54–57].

Whisker segments provide a powerful temporal record of the use of beach versus terrestrial resources because the δ^13^C and δ^15^N values of these sources are distinct in either one or both elements [7,21,54,56]. The C3 photosynthetic pathway, characteristic of most terrestrial vegetation on Santa Rosa and Santa Cruz Islands, produces δ^13^C isotope signatures in vegetation and the terrestrial food web that are well separated isotopically from the values of beach invertebrates that rely on kelp or plankton-based photosynthetic production [20,21,this study]. Furthermore, stable isotopes can provide information on the use of marine carrion by terrestrial mammals [7,21]. The sustained foraging by island fox on pinnipeds or their carcasses should be evident from ™^15^N of fox tissues; pinnipeds are high trophic level pelagic predators with ^15^N enriched isotope values [7,58].

Whiskers were collected from individual adult foxes from the three beach sites, Soledad (n = 10 adult foxes, December 1–3, 2018), Arlington Springs (n = 5 adults, October 27–29, 2018), and China Camp (n = 8 adults, December 12–14, 2018) (Fig 1). Soledad and Arlington Springs beach sites are located at opposite ends of a single beach system separated by a rocky outcrop, with the eastern-most trap station at Arlington Springs approximately 760 m from the western-most trap station at Soledad. At each site, 12 single-door, wire-welded traps (23 by 23 by 66 cm, Tomahawk Live Trap Co., Tomahawk, WI) were baited with dry cat and dog food and lured with Trapper’s Choice loganberry paste and deployed haphazardly along the beach-upland ecotone and adjacent upland overlapping scat collections sites. Sites used for whisker collection were chosen based on proximity to permitted monitoring grids and variation in the abundance of kelp wrack and endemic beach fauna (see Results).

Traps were deployed for three consecutive 24-hour periods and checked once a day, in the morning. Captured foxes were restrained without anesthesia and scanned for a passive integrated transponder (PIT) tag for individual identification. If no PIT tag was present, one was inserted subcutaneously between the scapulae as part of the Channel Island National Park island fox monitoring program. Data were recorded on sex, weight, age class (based on tooth wear), body condition (scale of 1–5) and reproductive status (not presented here). Body condition was assessed qualitatively for each fox as: (1) emaciated, starving, (2) slim, (3) considered a “normal, healthy, wild fox”, (4) has extra fat layer, and (5) obese. Foxes’ weight (to the nearest 10 g) was determined using a Pesola digital hanging scale by subtracting the weight of the trap from the weight of the trap with the fox.

We collected two whisker samples by plucking the longest whiskers from both the left and right side of the snout with tweezers. Whiskers were stored in paper envelopes at room temperature until processed for stable isotope analysis. One whisker was processed and the second whisker archived. Data from pups (0 age group) were not included since weaning might affect the N isotope values of pups [59,60]. All trapping and handling procedures followed the guidelines of the American Society of Mammologists [61], and sample collection was approved under National Park Service Research and Collecting Permit #CHIS-2018-SCI-0007.

### Isotope analysis of fox whisker segments

In the laboratory, individual fox whiskers were rinsed in chloroform-methanol (2:1) to remove oils and other contaminants [62,63], rinsed in deionized water, and dried at 60°C overnight. Dried whiskers were weighed and the length measured, and then sectioned using a razor blade from the proximal to the distal end into segments weighing approximately 200–300 μg each. Each segment was individually labeled to identify its position along the length of the whisker, weighed on a Cahn microbalance and packed into a tin capsule for stable C and N isotope analysis. Total whisker length varied from 5.8 to 7.9 cm. Unfortunately, there are no published data on the growth rate of island fox whiskers. If we assume whisker growth rate is comparable to that of another canid, wolves (*Canis lupus*) of ~0.04 cm day^-1^ [64], an assumption made for red fox [57], one whisker yields a dietary record spanning ~145 to 198 days (4.8 to 6.6 months). For the Soledad and China Camp sites where whisker samples were collected in early and mid-December 2018, this represents a dietary record from approximately July through December 2018. For Arlington Springs, where whisker samples were collected at the end of October 2018, this represents dietary record from May through October 2018.

Stable C and N isotope analysis of the whisker segments was conducted in the Marine Science Institute Analytical Laboratory at the University of California, Santa Barbara using a ThermoFinnigan DELTAplus Advantage continuous flow isotope ratio mass spectrometer interfaced with a Costech EAS elemental analyzer. Instrument precision, as standard deviation, determined from replicate analyses (n = 16) of the same standard (L-glutamic acid USGS40, δ^13^C = -26.39, ™ ^15^N = -4.52) was ± 0.10‰ for ^13^C and ± 0.13‰ for ^15^N. The natural abundances of carbon and nitrogen isotopes in each whisker segment are expressed in standard δ notation and calculated as follows for element *X*: δ^*n*^*X* = 1000 x ((R_*sample*_−R_*standard*_)/R_standard_), where *R* = ^n^*X*/^*n-1*^*X* expressed per mil (‰) relative to the Pee Dee Belemnite (PDB) standard for carbon and atmospheric N_2_ for nitrogen. Measured δ^13^C and δ^15^N values were corrected for signal strength bias, drift, and normalized using multipoint linear regression.

### Isotopic niche width

We used Stable Isotope Bayesian Ellipses in R (SIBER) [65] to visually and quantitatively compare individual and population variation in isotopic niche width, a proxy for diet breadth that represents both organic matter sources through variation in δ^13^C and δ^15^N values, and trophic position, primarily through variation in δ^15^N values [54,65,66]. Isotopic niche width was calculated using the individual whisker segment data for each fox and thus incorporates temporal variation in diet. This metric was only compared among individuals and populations of foxes from Soledad and China Camp, which were sampled during approximately the same time period, using the corrected Standard Ellipse Area (SEA_C_) and the Bayesian Standard Ellipse Area (SEA_B_). Because the length of sampled whiskers, and thus number of segments, varied among foxes, we standardized across individuals by using only the most proximal eight segments in the SIBER analysis.

To place the δ^13^C and δ^15^N values of fox whisker segments into isotopic context relative to potential beach and terrestrial foods, we also present values measured in this study or from the literature for selected taxa found in fox scats, including the talitrid amphipod, *Megalorchestia* from Soledad and China Camp (™^13^C = -14.7 ± 1.1, ™ ^15^N = 11.2 ± 0.4‰, n = 3 and ™^13^C = -14.9 ± 0.3, ™ ^15^N = 10.5 ± 0.1‰, n = 3, respectively, x ± 1SD, whole animal, this study), deer mouse muscle (*Peromyscus maniculatus*, from SCI, ™^13^C = -23.7 ± 0.5, ™ ^15^N = 5.6 ± 0.7‰, n = 3, [67]) and deer mouse hair in scats from Soledad Beach, SRI (-22.5 ± 0.8, 9.8 ± 2.2‰, n = 9, this study), pinniped average values corrected to keratin (*Zalophus californianus*, *Phoca vitulina*, *Mirounga angustirostris*, from central California, -15.6 ± 0.8, 16.5 ± 1.0, n = 101, [7], pelagic red crab (*Pleuroncodes planipes*) from southern California (-18.7 ± 0.2, 14.0 ± 0.7‰, n = 5, [68]), and Jerusalem cricket (*Stenopelmatus fuscus*) from SCI (-26.3 ± 0.9, 5.8 ± 0.5, n = 6, [67]. For presentation, mean isotope values of potential foods were adjusted upward to account for the expected isotopic enrichment of fox whisker tissue relative to food using the mean trophic discrimination factors (TDFs) from a feeding experiment in which the isotopic values of food items fed to captive red fox were measured together with their whiskers (Δ^13^C = 2.6 ± 0.1‰, Δ ^15^N = 3.4 ± 1.2‰, mean ± 1SD, [69]).

## Results

### Spatial variability in beach invertebrates

Here, we focus on variability among beaches in the abundance and biomass of those upper beach invertebrate taxa most prevalent in fox scats, talitrid amphipods, beach isopods, and the beetle *Thinopinus pictus* (see below), but also include the lower beach-swash zone inhabiting sand crab, *Emerita analoga*, which were abundant, but only recorded from one scat.

**Fig 2:**
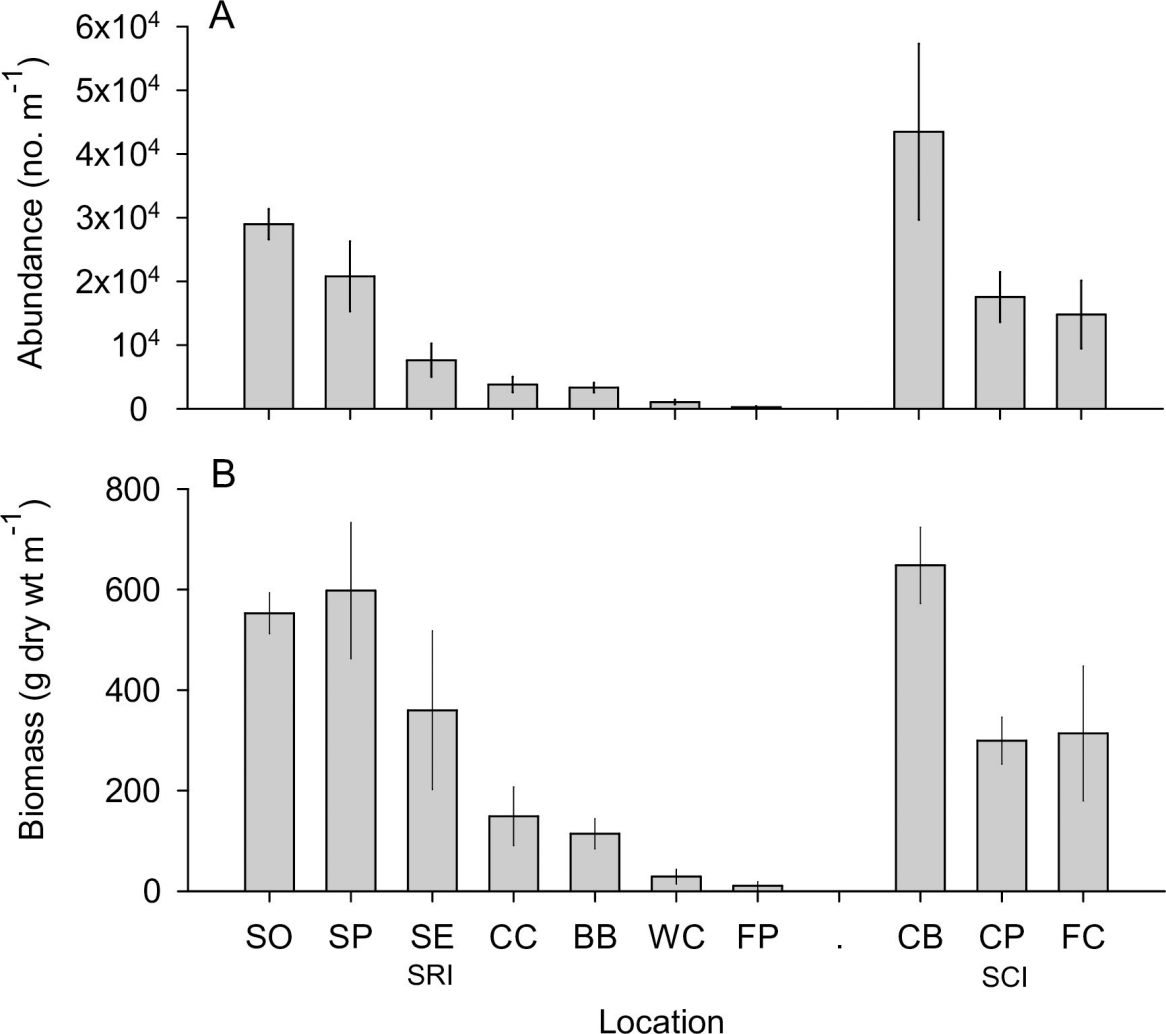
Abundance and biomass of upper beach and wrack associated invertebrates across beaches. (A) Abundance and (B) biomass values for talitrid amphipods, *Megalorchestia* spp., isopods *Alloniscus perconvexus* and *Tylos punctatus*, and staphylinid beetle *Thinopinus pictus* combined for each study beach. Mean ± SE, n = 5 (Santa Rosa Island) and n = 3 (Santa Cruz Island) transects. Site abbreviations as in Fig 1.

The abundance of combined wrack associated amphipod, isopod, and beetle taxa varied over 1000-fold (*p* < 0.001, *F* = 23.892, *df* = 9, one-way ANOVA) and biomass over 100-fold (*p* < 0.001, *F* = 13.356, *df* = 9) across beaches (Fig 2A and 2B). *Post hoc* pairwise comparisons revealed that Soledad, Sandy Point and Southeast Anchorage beaches on Santa Rosa Island and the three beaches on Santa Cruz Island had significantly higher abundances of these invertebrates than Bechers Bay, Ford Point and Water Canyon (*p’s* < 0.05, Šidák multiple comparison tests, S3 Table), with China Camp intermediate between those two groups. A generally similar pattern was evident for biomass with Soledad, Sandy Point, Southeast Anchorage on Santa Rosa Island, and all three beaches on Santa Cruz Island having the highest biomass, and Ford Point and Water Canyon the lowest biomass (*p’s* < 0.05, Šidák test, S3 Table).

Although only one island fox scat (from Southeast Anchorage, SRI) contained the remains of the lower beach-swash zone suspension feeding sand crab, *Emerita analoga*, this crab was ubiquitous, with mean abundance varying 20-fold (*p* = 0.001, *f* = 4.302, *df* = 4, 32, One-way ANOVA) and biomass 70-fold across beaches (*p* < 0.001, *f* = 14.524, *df* = 4, 32, note: Levene’s test for biomass, *p* = 0.02) (S4 Table).

### Carcasses and live pinnipeds

Carcasses of pinnipeds (sea lion, *Zalophus californianus*) and birds (shearwater spp., Procellariidae) were observed on five of 10 beaches during our surveys in 2018 (S5 Table). The carcasses were old and desiccated except for a sea lion at Coches Prietos, SCI, which was decayed, but intact and probably had been deposited within a few days prior to our visit, the apparent victim of a shark attack. The beach most heavily used by live pinnipeds in 2018 was China Camp with 63 northern elephant seals (*Mirounga angustirostris*) recorded during our survey. This beach is an important haul out and molting location for these animals during the fall months, and molted skin fragments with hair were common in the middle to upper beach.

Over three years (2016–2018) of annual surveys, we recorded carcasses at all but one site (Bechers Bay). Carcasses recorded during 2016 and 2017 included pinniped (northern elephant seal, sea lion), bird (western gull, *Larus occidentalis*, cormorant, *Phalacrocorax* sp.), and invertebrate (pelagic red crab, *Pleuroncodes planipes*) remains. Across the three years, most pinniped and bird carcasses observed were old (25 of 28, excluding invertebrates). The beach most heavily used in 2016 and 2017 by live pinnipeds was also China Camp with over 250 elephant seals recorded in each of those years (S5 Table).

### General diet composition

Upper beach invertebrates, crustaceans and insects, were present in at least some fox scats from all sites (S2 Table). The most prevalent arthropods were beach-dwelling talitrid amphipods (*Megalorchestia* spp.), present in scats from nine of 10 sites, the flightless pictured rove beetle, *Thinopinus pictus*, a predator of *Megalorchestia* [50] (seven of 10 sites), and beach-dwelling isopods (*Alloniscus perconvexus*, *Tylos punctatus*) (seven of 10 sites). The pelagic red crab *Pleuroncodes planipes*, washed onto the beach as carrion, was present in a few scats from three sites on SRI (S2 Table). Anthropogenic material (plastic fishing lures) was present in a few scats from three of 10 sites.

Terrestrial food material was present in some scats from all sites and included insect, deer mice, bird, and plant remains (S2 Table). Common insects included Jerusalem crickets (*Stenopelmatus fuscus*) (10 of 10 sites) and grasshoppers (Orthoptera) (10 of 10 sites) along with earwigs (Dermaptera) (six of 10 sites), terrestrial beetles (Tenebrionidae) (seven of 10 sites) and isopods (*Porcellio*) (three of 10 sites). The remains of deer mice *Peromyscus maniculatus* (hair, bones) were present in some scats from all sites as were fruits (manzanita (*Arctostaphylos* sp.), Australian saltbush (*Atriplex semibaccata*), toyon (*Heteromeles arbutifolia*)) along with leaves and unidentifiable plant material (S2 Table).

### Fox scat diet metrics and spatial variability in beach invertebrates

**Fig 3:**
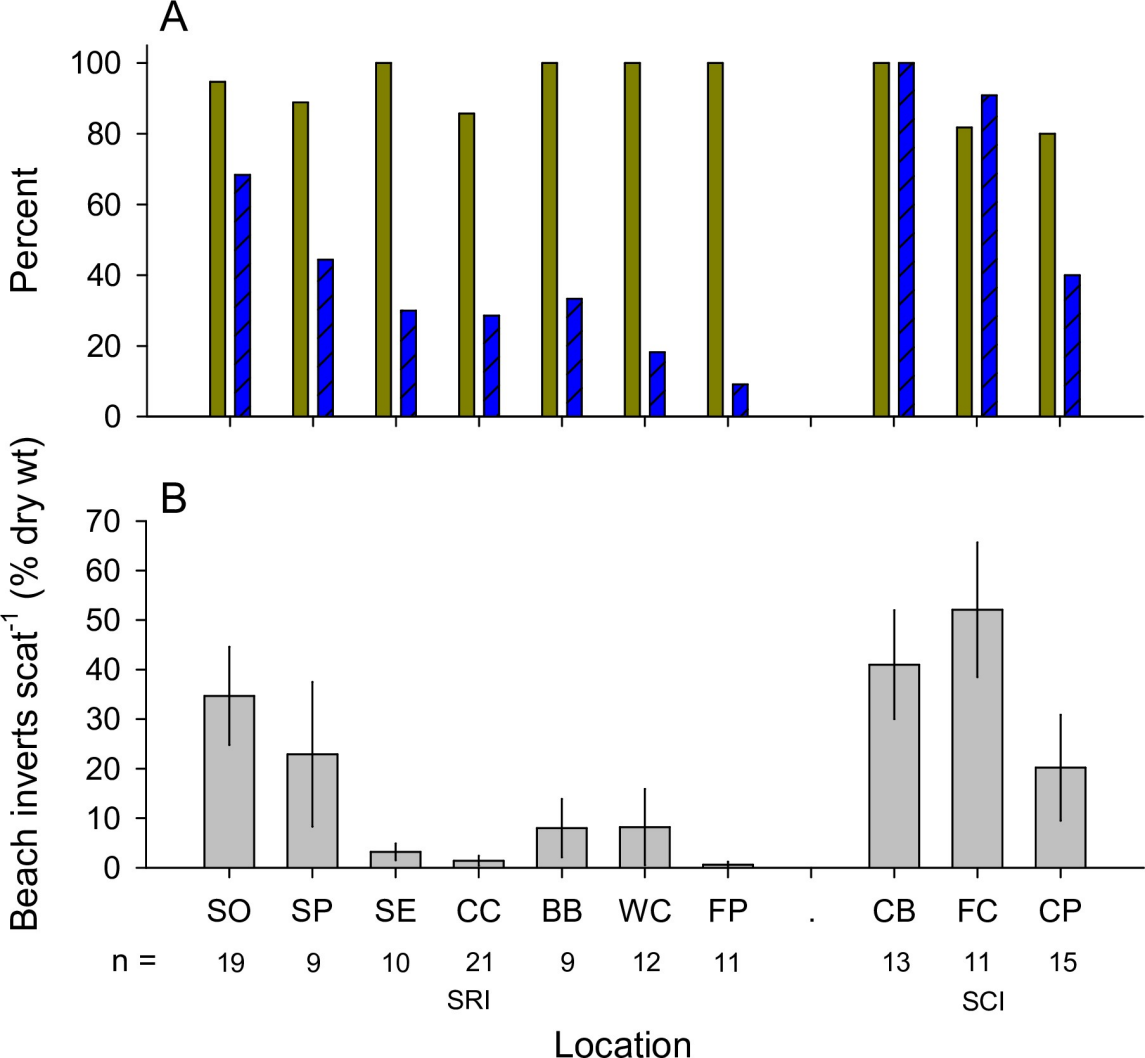
Contribution of beach material to island fox scat. (A) Proportion (as percent) of island fox scat containing terrestrial (solid brown) and endemic beach (hatched-blue) material, (B) mean percent (± SE) of individual scat (by dry weight) consisting of beach material from each beach. Site abbreviations as in Fig 1.

**Fig 4:**
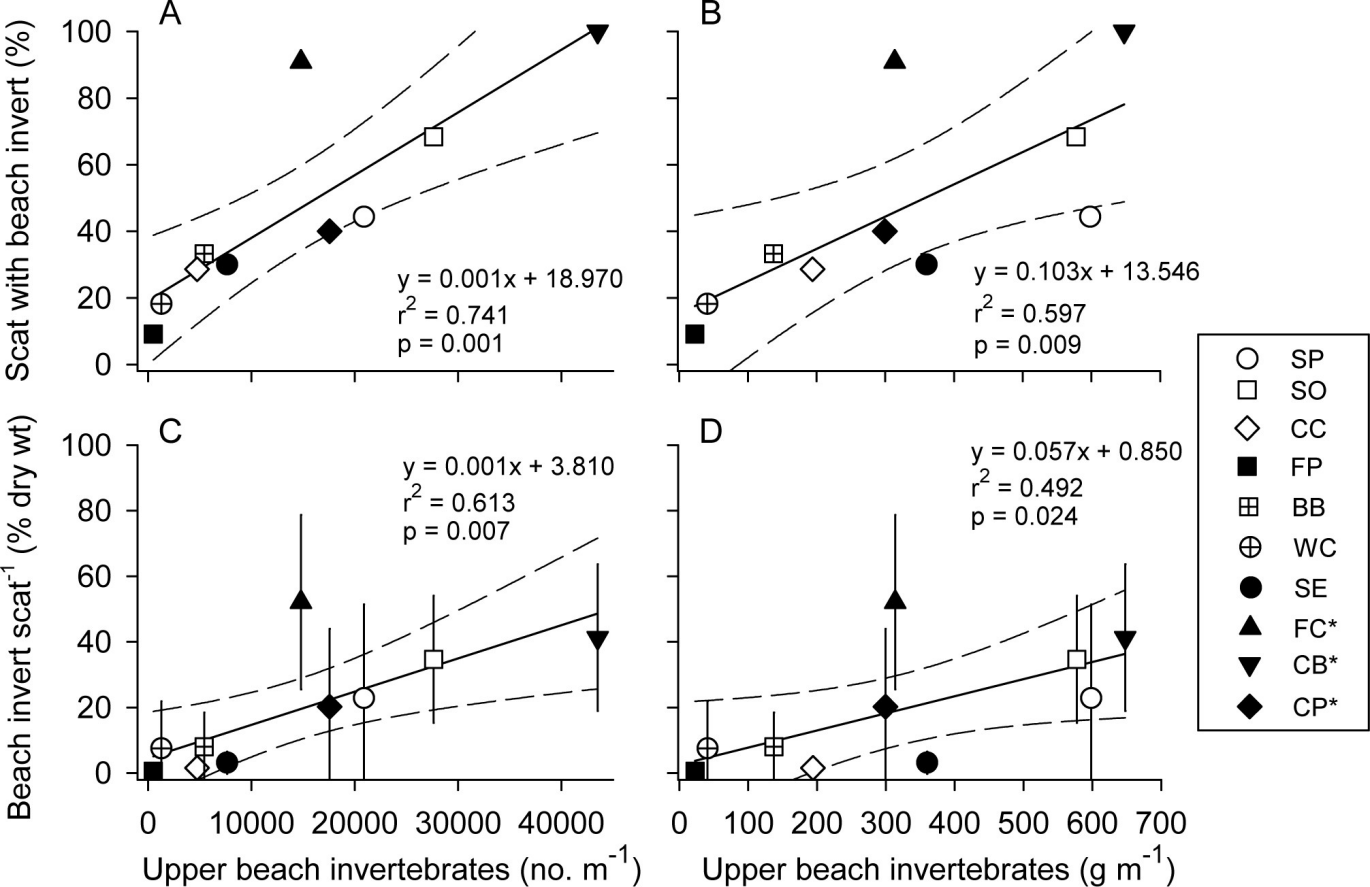
Relationships between beach material in scats and endemic beach invertebrate abundance and biomass. Proportion (as percent) of scat with endemic beach invertebrates and (A) invertebrate abundance and (B) biomass, and mean percent of invertebrates per gram scat versus (C) invertebrate abundance and (D) biomass at Santa Rosa and Santa Cruz Island study sites. Mean values for (C) and (D) ± 95% confidence intervals. Regression fits weighed for differences in sample size among sites. Dashed lines show 95% confidence intervals around regression lines. Site abbreviations as in Fig 1. *Santa Cruz Island.

Scat analysis revealed that the use of beach resources by foxes, as percentage of scats with beach prey, varied significantly among sites (*p* <0.001, test statistic = 43.471, Fisher’s Exact Test) (Fig 3A) and increased with the combined abundance of upper intertidal amphipod, isopod, and beetle prey (*p* = 0.001, *r*^*2*^ = 0.741, Fig 4A). The percentage of scats with beach prey also increased with the biomass of this prey (*p* = 0.009, *r*^*2*^ = 0.597, Fig 4B).

The percentage of beach prey by weight in individual fox scats also differed among beaches (*p* < 0.001, *H* = 45.822, *df* = 9, Kruskal-Wallis test), ranging from 0 to 52% (Fig 3B). Mean values increased significantly with the abundance of amphipod, isopod, and beetle prey (*p* = 0.007, *r*^*2*^ = 0.613, Fig 4C). Percentage of beach prey by weight also increased with the biomass of this prey (*p* = 0.024, *r*^*2*^ = 0.492, Fig 4D). Individual scats contained up to 69 *Megalorchestia*, 67 beach isopods, and 26 *Thinopinus*.

### Drivers of the abundance and biomass of invertebrate prey

In DistLM analysis, the abundance of *Macrocystis* wrack (cover) explained a significant (*p* = 0.019) amount (60%) of variation in the abundance of talitrid amphipods and *Thinopinus* beetles across beaches (Table 2). Similarly, *Macrocystis* wrack cover explained a significant (*p* = 0.003) amount (75%) of variation in amphipod-beetle biomass across beaches with beach (north-south) orientation (*p* = 0.016) explaining an additional 13% of variation in the data (Table 2). However, cover of *Macrocystis* did not explain a significant amount of variation in the abundance or biomass of beach isopods, the second most abundant beach prey. For these taxa, only beach orientation (east-west) explained a significant amount of variation and only for isopod biomass (37%, *p* = 0.026) across sites (Table 2). The other measured variables were not significant predictors of the abundance or biomass of talitrid amphipods or beach isopods in the DistLM models. Correlation analysis revealed that the cover of *Macrocystis* wrack on the beach was not associated with offshore kelp canopy biomass (*r* = 0.481, *p* = 0.16).

**Table 2:** Results of DistLM analysis evaluating relationships between the abundance and biomass of endemic beach invertebrates and predictor variables.

A. *Megalorchestia* & *Thinopinus*		Abundance (no. m^-1^)			
Predictor variable	Adjusted R^2^	Pseudo-F	P	Prop.	Cumul.
*Macrocystis* (cover)	0.550	12.011	0.019	0.600	0.600
		Biomass (g m^-1^)			
*Macrocystis* (cover)	0.717	23.767	0.003	0.748	0.748
Orientation (north-south)	0.848	7.925	0.016	0.134	0.882
B. *Alloniscus* & *Tylos*		Biomass (g m^-1^)			
Orientation (east-west)	0.286	4.607	0.0256	0.365	0.365

Abundance and biomass of (A) talitrid amphipods (*Megalorchestia* spp.) and beetle *Thinopinus pictus*, and (B) beach isopods (*Alloniscus perconvexus*, *Tylos punctatus*), evaluated against predictor variables of giant kelp (*Macrocystis pyrifera*) cover, beach length, width, slope, and orientation, and presence/absence of dunes. Only significant relationships (*p* < 0.05) shown.

### Stable isotope analysis of fox whisker segments

**Fig 5:**
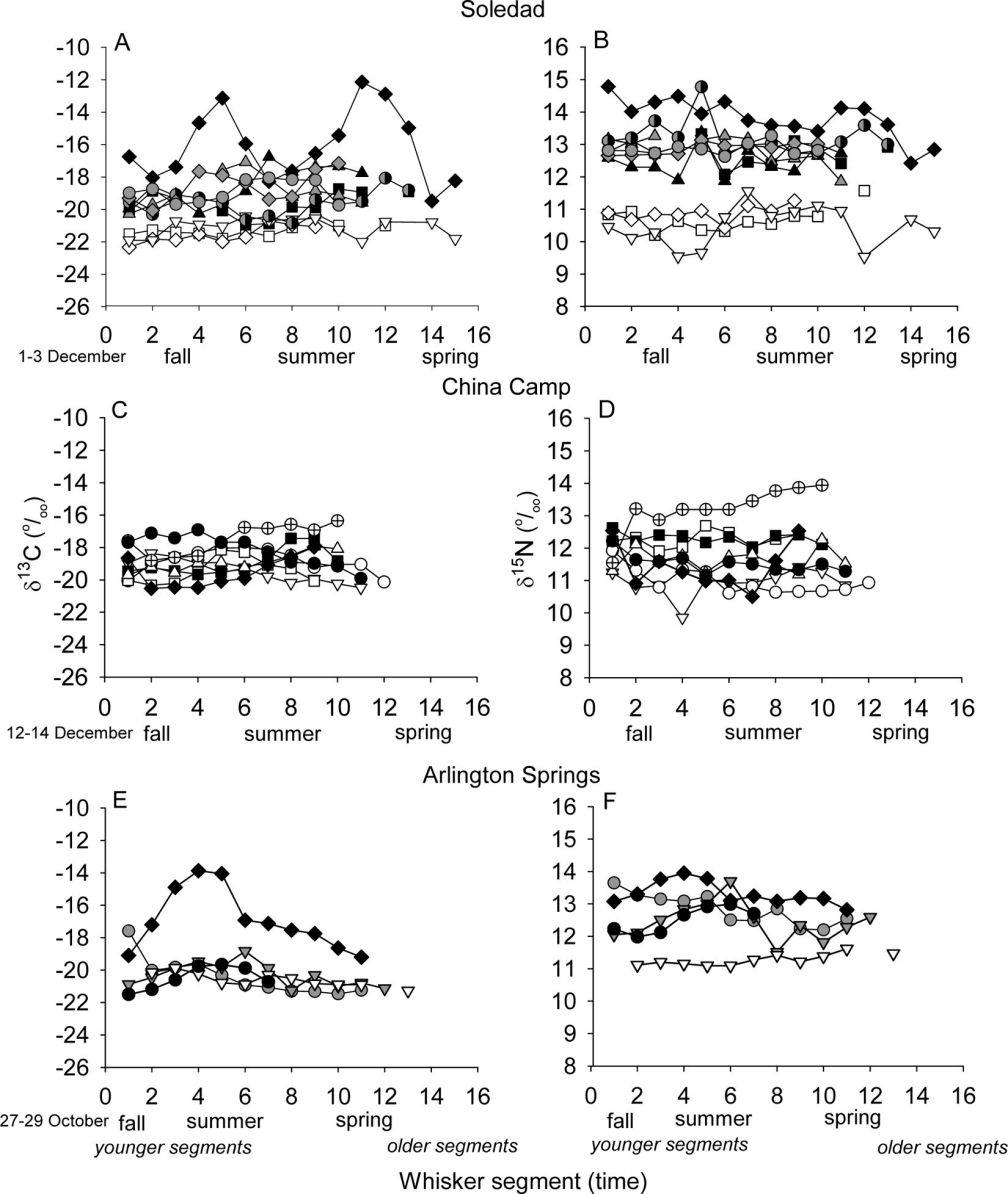
δ^13^C and δ^15^N values of island fox whisker segments. Values from individual foxes trapped at (A, B) Soledad (n = 10 foxes), (C, D) China Camp (n = 8 foxes), and (E, F) Arlington Springs (n = 5 foxes). Date of whisker collection provided in lower left of each figure pair. Whiskers varied in length and therefore number of segments. Segment 1 is most proximal to the face. Each segment represents an approximate two week time period.

**Fig 6:**
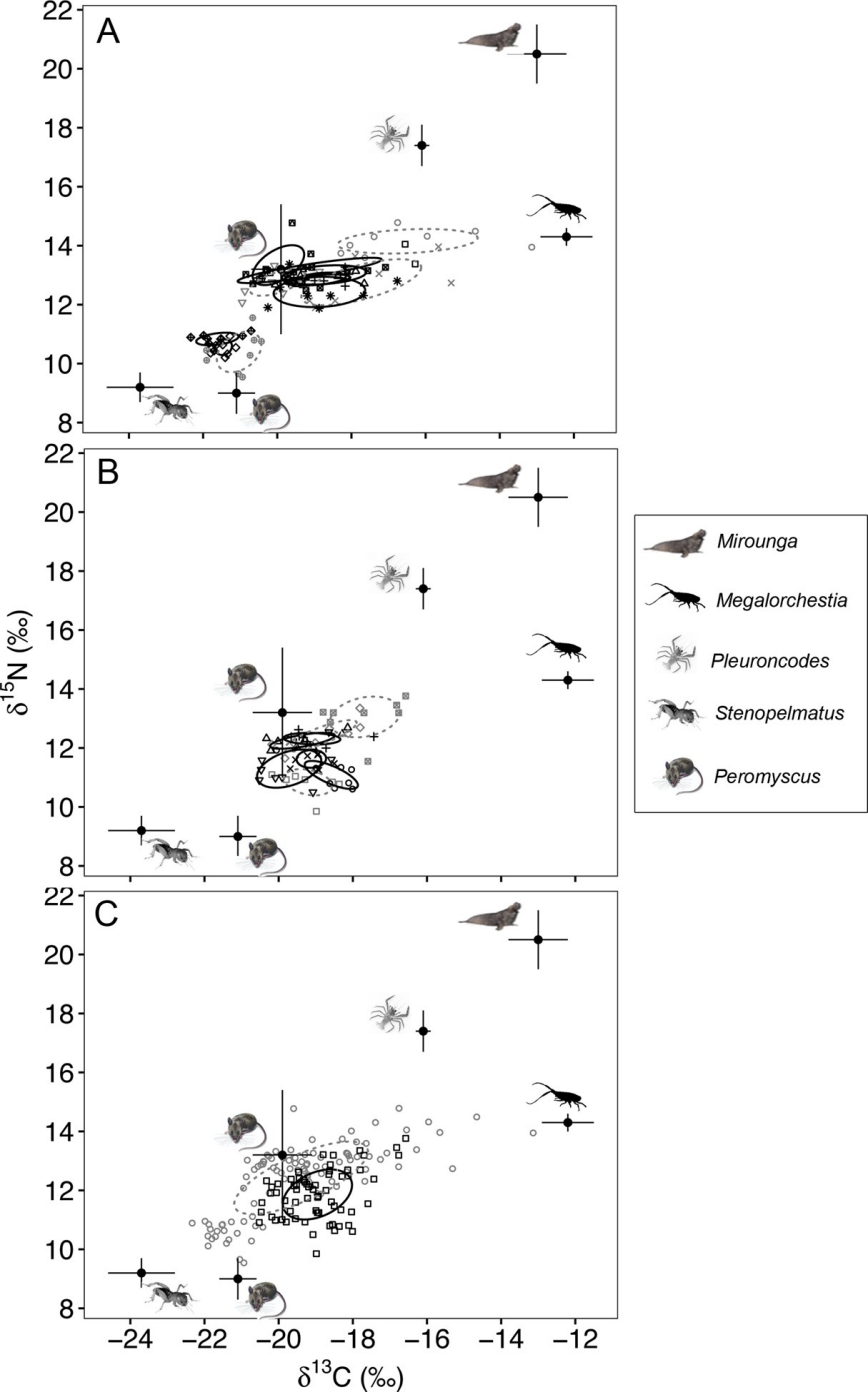
Bivariate plots of island fox whisker segment δ^13^C and δ^15^N values for Soledad and China Camp beaches. (A) Soledad and (B) China Camp beaches, and (C) comparison of populations from the two sites. The two beaches were sampled within two weeks of each other. Isotopic niche width visualized as standard ellipses enclosing 40% of the isotope space for individual foxes and for populations at each site. Dashed gray lines enclosing values for males, solid black lines enclosing females. Standard ellipse areas (SEA_C_ and SEA_B_) for individuals and each population are given in Table 3. Mean δ^13^C and δ^15^N values of potential foods, included for context, are adjusted upward to account for expected trophic discrimination between fox and food (TDFs: Δ^13^C = 2.6‰ and Δ^15^N = 3.4 ‰).

The exploitation of beach foods by individual foxes at Soledad, Arlington Springs, and China Camp was supported by the δ^13^C and δ^15^N values of whisker segments (Figs 5 and 6). These data suggest that the nutritional importance of beach resources varied among individuals, over time, and between Soledad and China Camp populations.

Three dietary groups were evident at Soledad based on temporal variation in individual whisker segment δ^13^C and δ^15^N values (Fig 5A), and further distinguished in isotope bivariate plots (Fig 6A). Three of 10 foxes had generally low δ^13^C (-22.0 to -20.2‰) and δ^15^N (9.5 to 11.6‰) values, consistent with a diet of largely terrestrial foods. Seven of 10 foxes had higher isotope values with δ^13^C (-20.8 to -16.8‰) and δ^15^N (11.9 to 14.8‰) values suggesting a mixed diet including beach foods, while one fox (solid diamond) had elevated, but variable δ^13^C (-19.5 to -12.1 ‰) and δ^15^N (12.4 to 14.8‰) values suggesting more intense use of beach foods (Fig 6A).

Two distinct peaks in δ^13^C segment values were present for this fox, suggesting increased importance of ^13^C enriched beach foods during two approximate four to six week periods in the fall-summer (assuming the time interval between segments is approximately two weeks, see Materials and Methods). δ^15^N values were also elevated during these periods, but not to the extent expected from sustained foraging on pinniped [7] or seabird [67] carcasses (i.e., > 18 ‰, corrected for trophic discrimination).

Though temporally out of phase with Soledad (and China Camp) by approximately two months, isotopically distinct groups were also distinguishable at Arlington Springs, which is contiguous with the Soledad beach site (Fig 5). One fox (solid diamond) had elevated values (δ^13^C, -19.2 to -13.8 ‰; δ^15^N, 12.8 to 13.9‰) in late summer-fall suggesting increased importance of ^13^C enriched beach foods, while four foxes had lower values (δ^13^C, -21.5 to -18.8 ‰; δ^15^N, 11.1 to 13.7‰), suggesting more reliance on terrestrial foods.

Whisker isotope data from China Camp also suggested some use of beach foods (δ^13^C, -20.0 to -16.4 ‰, δ^15^N, 9.9 to 13.9 ‰), but with more overlap among foxes and without the pronounced peaks in δ^13^C values evident at Soledad and Arlington Springs. One fox with elevated δ^13^C and δ^15^N values in more distal segments may have been using beach foods more than the others during that period. In no case did δ^15^N values of whisker segments from these sites approach the highly elevated values expected to result from the sustained foraging on pinniped or seabird carcasses.

### Isotopic niche width, individual specialization, and fox performance metrics

Standard Ellipse Areas (SEA_C_ and SEA_B_) varied over 10-fold among individuals within the Soledad and China Camp populations (Table 3 and Fig 6). All foxes could be construed as dietary “specialists”; individual SEA values were much smaller than the overall population values for each site [57,63,70,71], which together with position in isotope space, reflected individualism in use of beach and terrestrial foods (Fig 6). The SEA values for the fox population at Soledad was nearly twice that of the population at China Camp, consistent with inclusion of individuals specializing on terrestrial or beach foods at this site (Fig 3).

**Table 3:** Standard ellipse area (SEA_C_ and SEA_B_) from SIBER analysis using whisker segments.

Site	Fox no.	Sex	SEA_C_ (‰^2^)	Population SEA_C_ (‰^2^)	SEA_B_ (‰^2^)	Population SEA_B_ (‰^2^)	Body wt (kg)	Condition index
Soledad	80155	F	0.20	4.63	0.15	4.58	2.52	4
	79892	F	0.37		0.29		2.6	4
	UNK	F	0.44		0.34		na	na
	79743	F	0.50		0.41		2.5	4
	80030	M	0.98		0.76		3.03	5
	80089	F	1.15		0.89		2.74	4
	79607	M	1.29		1.03		2.51	4
	79636	F	1.33		1.08		2.71	4
	26199	M	2.11		1.69		2.69	4
	01852	M	2.53		1.99		2.5	3
China Camp	79756	F	0.40	2.42	0.31	2.33	1.71	3
	61904	M	0.50		0.39		2.35	3
	58012	F	0.59		0.50		1.88	3
	80296	F	0.68		0.55		2.21	3
	37547	F	0.77		0.66		na	na
	37503	M	0.92		0.73		2.7	4
	A2B2C	F	1.71		1.36		1.83	3
	23989	M	2.11		1.67		2.94	3

SEA values of individuals and populations of island fox trapped in December 2018 from Soledad and China Camp beach sites together with body weight and conditon of foxes from each site. Analysis used the most proximal eight segments from each sampled whisker. na = not available.

**Fig 7:**
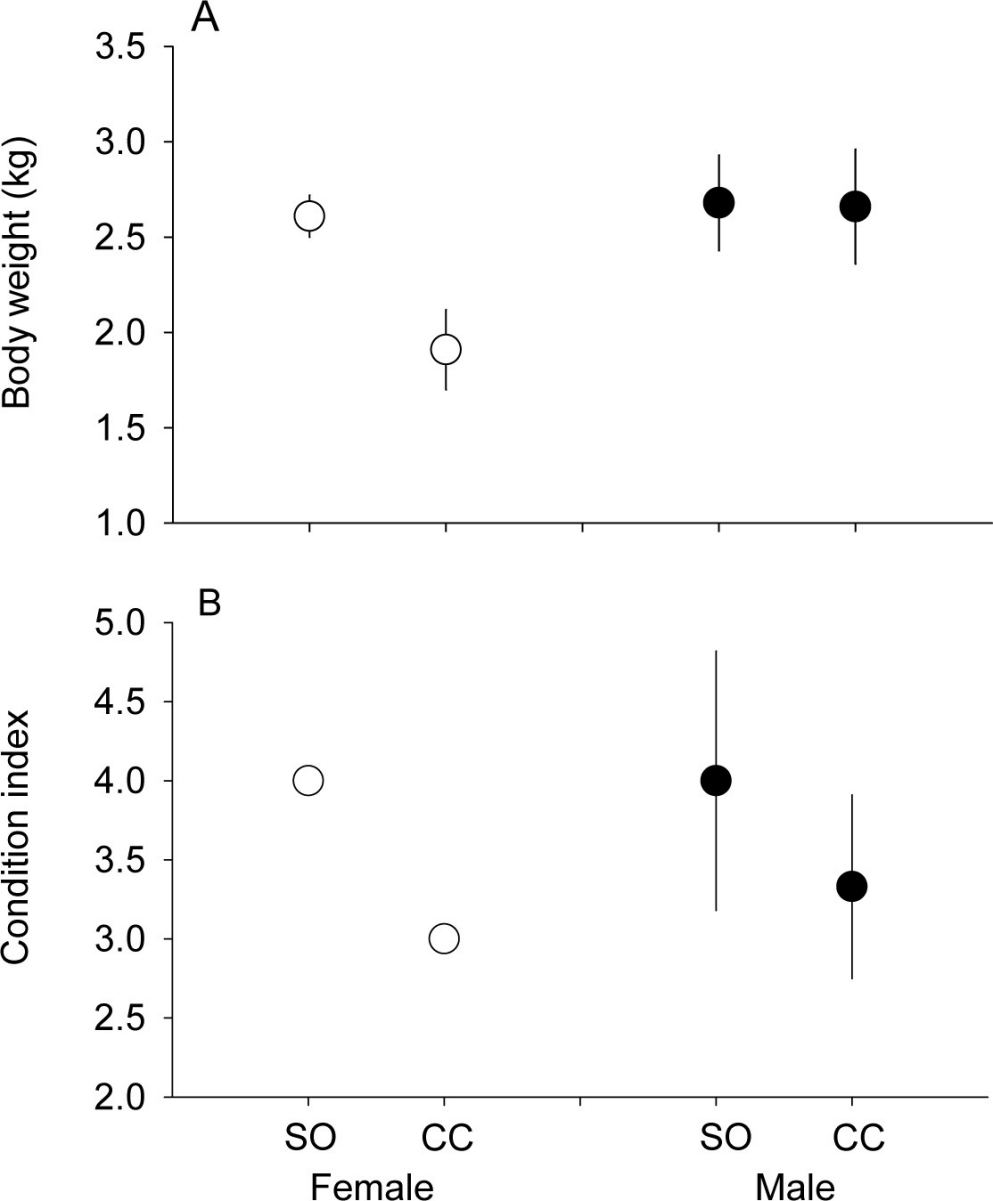
Comparison of (A) body weight (kg) and (B) condition index for female and male adult foxes between Soledad (SO) and China Camp (CC) locations. Mean ± SD.

There was a significant site by sex interaction on fox body weight (*p* = 0.008, *f* = 10.079, *df* = 1, 12, two-way ANOVA). Consequently, we evaluated the effect of site on body weight separately for males and females. Females weighed on average ~27% more at Soledad than at China Camp (*p* < 0.001, *t* = 2.992, *df* = 7, Student’s test, Fig 7A), but there was no difference in mean body weight for males between sites (*p* = 0.786, *t* = 0.082, *df* = 5). There was a significant effect of site, but not sex on fox condition index (site, *p* = 0.005, *f* = 12.097, *df* = 1,13, sex, *p* = 0.50, *f* = 0.484, *df* = 1, 13, sex x site, *p* = 0.50, *f* = 0.484, *df* = 1, 13 two-way ANOVA) (Fig 7B). Mean individual SEA varied significantly between males and females, but not between Soledad and China Camp beaches (sex, *p* = 0.03 for SEA_C_ and SEA_B_, *f’s* = 5.81, 5.54, site, *p’s* = 0.5, *f’s* = 0.436, 0.384, site x sex, *p’s* = 0.2, *f’s* = 0.241, 0.228, *df’s* = 1,14).

## Discussion

### Use of beach resources by island fox

Analysis of scat collected across beach to nearshore upland habitat on Santa Rosa and Santa Cruz Islands revealed that island foxes were foraging across these habitats on a variety of terrestrial foods including fruits, deer mice, insects, and birds consistent with the diet analyses in other studies [11,17–19,72]. Our results differ from previous studies in showing substantial, although variable use of endemic beach invertebrate prey, the most prevalent of which were upper beach, wrack-associated talitrid amphipods (*Megalorchestia* spp.). There was little evidence for use of the widespread lower beach-swash zone sand crab, *Emerita analoga*, by fox (only one crab found in one scat) despite the high abundance of these suspension feeders at most beaches. These highly mobile crabs burrow in wet sand with only their long feathery second antennae extended to capture planktonic food from the wave wash. Foraging for live sand crabs would require digging into the wet lower beach or swash zone that is frequently affected by wave wash, which could discourage island fox from the greater use of this abundant potential resource. Foxes also fed on non-beach marine invertebrates stranded as carrion, including the pelagic red crab (three sites) and unknown brachyuran crabs (four sites), but no visible remains (skin, hair) of marine vertebrate carrion were observed in scats.

Studies of island fox diet have generally reflected scat collection across the larger terrestrial landscape, and although beach foods were poorly represented in scats in previous work, island fox were shown to exploit these resources. Phillips et al. [18] found terrestrial arthropods, vertebrates, and plants in fox scats collected in grass, scrub or mixed habitats on San Clemente Island and decapod crab remains were found in 31 of 476 scats (6.5%) in one year (1993). Cypher et al. [19] found pinniped remains in trace amounts (< 5% of scats from San Nicholas, San Miguel, San Clemente, Santa Catalina Islands), and crustacean remains (*Megalorchestia californiana*, *Emerita analoga*, unidentified crabs) in ≥ 10% fox scats in the summer-fall (2009) on San Clemente and in the summer (2009) on Santa Rosa Island, indicating that foxes foraged along the shoreline. Our finding that upper beach and wrack associated crustaceans and insects were prevalent in island fox scats from beaches and adjoining upland suggests that this prey can be an important component of fox diet, but the use of this resource can vary substantially among beaches.

### Predation and spatial patterns in endemic beach invertebrates

Food availability and elements of the landscape can influence the resource value of habitat to foraging small mammals [24,73,74]. We found that the exploitation of upper beach invertebrates by island fox, as evidenced by remains in scats, was associated with prey availability, which varied dramatically, from 1000 to 100-fold in abundance and biomass, respectively, across the 10 study beaches. Given the use of beach prey, it follows that beaches with physical and biological characteristics favorable for invertebrate communities would provide the most productive foraging habitat for island foxes. These characteristics include the abundance of macroalgal wrack that provides food and habitat for beach invertebrates, and physical factors that could affect the delivery and retention of drift *Macrocystis* and other macrophyte wrack on the beach.

DistLM modeling revealed that the abundance of giant kelp wrack accounted for a significant, and the largest amount, of variation in *Megalorchestia*-*Thinopinus* abundance and biomass across all beaches. This finding is consistent with reports from mainland beaches, where the abundances of *Megalorchestia* can exceed fifty thousand individuals per meter of shoreline and correlate strongly with the amount of wrack on the beach [8,23,75]. The area of kelp canopy offshore of our study beaches in late summer-early fall 2018 (July, August, September), and the presumptive source of wrack to the beach inshore, did not account for a significant amount of variation in kelp wrack on the beach or the abundance or biomass of beach invertebrates. One possible explanation for this finding is that physical processes strongly influence the quantity of detached *Macrocystis* and other wrack that is transported to the beach. For example, Soledad and Sandy Point beaches with the highest wrack cover face northwest into the prevailing wind and currents [37] that may transport kelp onshore. In contrast, China Camp and Bechers Bay, which had offshore kelp beds comparable in size to those of Soledad and Sandy Point, but much lower wrack cover on the beach, face southwest and northeast, respectively (Table 1), where prevailing southeastward flowing currents may transport drift kelp away from rather than onto the shore. However, two beaches (Ford Point, Southeast Anchorage) with low abundances of wrack and beach invertebrates had both smaller kelp beds offshore and faced away from prevailing wind and currents (southeast, north-northwest, respectively), suggesting that multiple, potentially interacting factors could influence the quantity of kelp wrack on island beaches [31,76,77].

In contrast to our results for the amphipods, beach isopods, an appreciable component of fox scats from one beach (Coches Prietos, SCI), were most influenced by factors other than wrack abundance. *Alloniscus perconvexus*, the most common species in our samples, occurs higher on the beach than *Megalorchestia* and may be more catholic in their diet, feeding on drier kelp and on a broader range of food types [78]. It is possible that the abundance of beach isopods may be more related to physical features of the habitat, as postulated for these taxa on mainland beaches, but only beach orientation explained a significant amount of variation, and only in isopod biomass in our study. Collectively, our results suggest that in the coastal zone, elements of the “beachscape”, including orientation of the beach with respect to wind and currents, abundant *Macrocystis* wrack, and the associated high abundances of upper beach invertebrates enhance foraging opportunities for island fox. In contrast, down current beaches with low wrack cover, such as Ford Point, may provide less optimal foraging opportunities for island fox.

### Isotopic evidence for the use of beach resources

While the scat analysis provided a snapshot of the use of beach foods by foxes across 10 beach sites, the isotope analysis of whisker segments from a subset of those sites indicated the sustained use of these foods over time (weeks, months) by at least two foxes. These foxes (one each from Soledad and Arlington Springs), in particular, appear to have derived a substantial portion of their nutrition from the beach, based on ^13^C and ^15^N-enriched values of whisker segments relative to potential terrestrial foods. One source of enriched values, supported by both scat and isotope data, were upper beach invertebrates, particularly talitrid amphipods, which feed on giant kelp and other macroalgal wrack [26,79] and have δ^13^C and δ^15^N values well separated from terrestrial sources. Island fox have been considered insectivorous [32] due to terrestrial arthropods, including Jerusalem crickets, grasshoppers, and beetles, comprising an appreciable portion of their diet (e.g., [18,19], this study). Thus, predation on beach arthropods, including talitrid amphipods, beetles, and isopods, is consistent with the importance of invertebrate prey in the diet of this small fox. In this regard, the use of beach arthropods closely resembles the omnivorous diet of another threatened insular canid, the Darwin’s fox (*Pseudalopex fulvipes*) of Chile, which has also been reported to feed on insects and beach invertebrates [80]. Deer mice (*Peromyscus maniculatus*), known to feed on beach arthropods [81], were found in island fox scats at all sites and could serve as a trophic intermediate between beach invertebrates and island fox. This possibility is supported by the enriched δ^13^C and δ^15^N values of mouse hair obtained from fox scats at Soledad beach compared with published values (Fig 7), and observations of mice under driftwood in the beach–dune ecotone.

A sustained reliance on pinniped or other ^15^N-enriched carrion by island fox was not supported by our isotope results, although some use of this source is expected based on previous studies [17,19]. Both living and dead pinnipeds were present at half of our sites in 2018, and beached carcasses were only absent from one site, Bechers Bay, over three years of surveys. However, most of these were mummified carcasses, largely skin and bones likely deposited months to years prior to our study. Snapshot sampling of scat is less likely to capture erratic or pulsed inputs of carrion, and digestible soft tissues may not be evident in scat samples. We expected to see a pinniped isotope signal (elevated δ^15^N values) in the whisker data from China Camp foxes, given the heavy use of this site by pinnipeds, particularly elephant seals. Pinnipeds typically have high δ^15^N values (> 15‰ for hair [7]), which when corrected for trophic discrimination (+3.4 ‰,), could lead to δ^15^N values of whisker segments approaching 17 to 18 ‰ if feeding on pinniped was sustained. However, the δ^15^N values of foxes from this beach were not elevated relative to fox values from Soledad, which lacked pinniped carcasses and live pinnipeds during our surveys in 2018 (and few in number in 2016, 2017; S5 Table), suggesting minimal use of this resource, at least during the fall and summer months.

### Temporal variability in the use of beach resources

Dramatic temporal shifts in diet were suggested from longitudinal increases and then decrease in δ^13^C values of whisker segments for two foxes foraging on Soledad and Arlington Springs beaches. δ^15^N values did not increase appreciably (~1–3‰) during this period, which was not consistent with increased use of living or dead pinnipeds, or other higher trophic level predators. ^15^N isotopic enrichment consistent with use of pinnipeds has been reported for some ancient island fox samples (San Nicholas) [21] and contemporary coyote samples from Año Nuevo, northern California [7]. On the other hand, ^13^C enrichment of whisker segments with coincident minimal ^15^N enrichment would be consistent with the increased use of wrack associated invertebrates, and could reflect the temporal dynamics of endemic beach invertebrate populations [82], or variable foraging patterns of the island fox. Variability in diet often reflects the types of foods most available to terrestrial generalists, including red fox [83,84], kit fox [56], gray fox and coyote [85].

### Individual specialization, isotopic niche width, and fox performance

Island foxes exhibited distinct individual differences in their use of beach and terrestrial resources. The use of a significantly narrower set of resources by an individual than the population as a whole is termed individual specialization [57,63,71,86,87]. All of the individual foxes at Soledad and China Camp had SEA values considerably smaller than the population values, illustrating that both populations were comprised of specialists with much narrower isotopic niches than the overall populations (“Type B generalist” of [70]), a similar pattern to that reported recently for red fox (*Vulpes vulpes*) populations in urban and rural landscapes [57]. The isotopic niche width, as SEA values, of the fox population at Soledad was nearly twice that of the population at China Camp. Populations containing individuals specializing on terrestrial or beach foods are likely to exhibit broader isotopic niche variation than populations foraging solely in terrestrial habitats because of the wide isotopic separation between marine and terrestrial organic matter sources [88–90]. Other terrestrial mammals with broad isotopic niche widths, reflective of the use of some marine foods, include artic foxes that feed on pinnipeds and seabirds [91,92], coyotes exploiting pinnipeds [7], and grey wolves exploiting salmon and marine mammals [93].

Studies of diet specialization in mammalian predators have revealed that the degree of individualism in diet can reflect resource availability and predator density, and can vary over time [63,66,71]. The mechanisms contributing to individualism in the diet of coastal island fox remain to be determined, but the isotope data suggest some partitioning of habitat among foxes using primarily terrestrial foods, a mix of beach and terrestrial foods and, at Soledad (and Arlington Springs), a larger contribution of beach foods, and may reflect the inclusion of beach resources within a home range or territory that borders the coastline.

Intraspecific competition for resources, related to fox density, may contribute to the larger isotopic niche width of the fox population at Soledad compared with China Camp [71,86,94]. Fox population data collected by Channel Island National Park at sites ~0.75 km inland from these beaches using a standardized trapping protocol over the past five years (2014–2018), suggest that foxes were 2–3 times more abundant in the vicinity of Soledad (21 unique foxes trapped in 2018, 16.8 ± 1.6 foxes trapped 2014–2018, x ± SE) than China Camp (8 foxes trapped in 2018, 7.6 ± 1.2 foxes trapped 2014–2018) (S6 Table). Endemic beach food resources were considerably more abundant at Soledad than China Camp, and fox body weight (females only) and condition were higher for this population. Since marine food resources are generally considered beneficial to terrestrial consumers and populations, especially when terrestrial resources are in short supply [5,6,95], beaches that provide productive foraging habitat may help support locally higher fox densities, population sizes, and body weights. The significantly larger isotopic niche space of males, together with a lack of difference in body weight for males between the two sites, could reflect a wider foraging range for males, allowing access to a larger variety of food types, than females. Females also have a large energetic investment in pup production each year and may be more sensitive to resource availability, but further study is needed to evaluate possible relationships between food resource availability (beach and terrestrial) and fox population dynamics.

## Conclusion

Although the use of allochthonous marine subsidies by terrestrial mammals has been widely reported, their importance beyond an opportunistic foraging resource is often poorly understood. Our results suggest that the threatened island fox uses sandy beach food resources, particularly arthropods that are sustained by kelp wrack. The use of these invertebrates by island fox varied with their abundance, which in turn depended mainly on the magnitude of allochthonous kelp wrack subsidies across beaches. Our findings show that proximity to beaches with abundant marine-based food resources increases dietary niche breadth of island fox, which could increase the resilience of fox populations during declines in the availability of terrestrial resources associated with cyclic or stochastic events, or longer-term climate change.

## Supporting information

S1 Table Results of regression analysis untransformed and following weighting by sample size and logit transformation of A) proportion of scats with beach material and B) proportion of beach material per scat versus the abundance and biomass of combined endemic beach invertebrates (Megalorchestia spp., Thinopinus pictus, Alloniscus perconvexus, and Tylos punctatus).
(DOCX)

S2 Table The proportion of scats from each study site that contained a particular food type (as percent). (DOCX)

S3 Table Results of Šidák post hoc pairwise comparison tests of invertebrate A) abundance and B) biomass between beaches on Santa Rosa and Santa Cruz Islands. SO—Soledad, SP—Sandy Point, SE—Southeast Anchorage, CC—China Camp, BB—Bechers Bay, WC—Water Canyon, FP—Ford Point, CB—Christy, FC—Forney’s Cove, CP—Coches Prietos. *Santa Cruz Island. Only values with significant differences (p < 0.05) shown).
(DOCX)

S4 Table Abundance and biomass of the lower beach/swash zone suspension feeding sand crab, *Emerita analoga*, at the study sites on Santa Rosa (n = 5 transects) and Santa Cruz Islands (n = 3 transects). Mean ± SE. *Santa Cruz Island.
(DOCX)

S5 Table Number of live pinnipeds, and carcasses of pinnipeds and birds, and estimates for large invertebrates, recorded on each beach (up to 1 km of beach for longer beaches) during annual surveys in August–September, 2016–2018. No fish were observed. Values in parentheses are number of fresh carcasses out of total, otherwise values represent old/desiccated carcasses. Blank cell = 0. Year of island fox scat collection (2018) in boldface. *Santa Cruz Island.
(DOCX)

S6 Table Numbers of island fox trapped at Channel Island National Park monitoring grids located inland of Soledad (Dry Canyon) and China Camp beaches. Schamel et al., unpublished.
(DOCX)

S7 Table δ^13^C and δ^15^N values of island fox whisker segments. CC = China Camp, SO = Soledad, AS = Arlington Springs. Segment 1 is the most proximal segment. n/d = no data.
(DOCX)
