# Supplementary material for: Diet of a threatened endemic fox reveals variation in sandy beach resource use on California Channel Islands
Source: PLoS One. 2021 Oct 28;16(10):e0258919. doi: 10.1371/journal.pone.0258919 (PMC8553077; doi:10.1371/journal.pone.0258919)
Supplement: S1 Table — Results of regression analysis untransformed and following weighting by sample size and logit transformation of A) proportion of scats with beach material and B) proportion of beach material per scat versus the abundance and biomass of combined endemic beach invertebrates (Megalorchestia spp., Thinopinus pictus, Alloniscus perconvexus, and Tylos punctatus). (DOCX) [file pone.0258919.s001.docx]

Table S1

| A. Percent of scat with beach material as a function of: | | | | |  | B. Percent of beach material in scat as a function of: | | | |
| --- | --- | --- | --- | --- | --- | --- | --- | --- | --- |
| Abundance (No. m^-1^) | | Biomass (g m^-1^) |  |  |  | Abundance (No. m^-1^) | | Biomass (g m^-1^) | |
| Untransformed | |  |  |  |  | Untransformed | |  | |
| r^2^ = 0.707 | | r^2^ = 0.530 | |  |  | r^2^ = 0.513 | | r^2^ = 0.425 | |
| y = 0.002x + 0.192 | | y = 0.097x + 15.328 | |  |  | y = 0.001x + 4.629 | | y = 0.052x + 2.557 | |
| p = 0.002 |  | p = 0.017 |  |  |  | p = 0.012 |  | | p = 0.041 |
|  |  |  |  |  |  |  |  | |  |
| Weighted by sample size | | |  |  |  | Weighted by sample size | | | |
| r^2^ = 0.741 | | r^2^ = 0.597 | |  |  | r^2^ = 0.613 | | r^2^ = 0.492 | |
| y = 0.002x + 0.200 | | y = 0.103x + 13.546 | |  |  | y = 0.001x + 3.810 | | y = 0.057x + 0.850 | |
| p = 0.001 |  | p = 0.009 |  |  |  | p = 0.007 |  | | p = 0.024 |
|  |  |  |  |  |  |  |  | |  |
| Logit transformation (y) | | |  |  |  | Logit transformation (y) | | | |
| r^2^ = 0.747 | | r^2^ = 0.514 | |  |  | r^2^ = 0.568 | | r^2^ = 0.482 | |
| y = 0.000x - 1.567 | | y = 0.005x - 1.715 | |  |  | y = 0.000x - 3.479 | | y = 0.005x - 3.772 | |
| p = 0.001 |  | p = 0.020 |  |  |  | p = 0.012 |  | | p = 0.026 |
|  |  |  |  |  |  |  |  | |  |
| Logit transformation (y) weighted by sample size | | | | |  | Logit transformation (y) weighted by sample size | | | |
| r^2^ = 0.762 | | r^2^ = 0.560 | |  |  | r^2^ = 0.606 | | r^2^ = 0.524 | |
| y = 0.000x - 1.564 | | y = 0.006x - 1.787 | |  |  | y = 0.000x - 3.601 | | y = 0.006x - 3.949 | |
| p < 0.001 |  | p = 0.013 |  |  |  | p = 0.008 |  | | p = 0.018 |
