# Supplementary material for: Diet of a threatened endemic fox reveals variation in sandy beach resource use on California Channel Islands
Source: PLoS One. 2021 Oct 28;16(10):e0258919. doi: 10.1371/journal.pone.0258919 (PMC8553077; doi:10.1371/journal.pone.0258919)
Supplement: S2 Table — (DOCX) [file pone.0258919.s002.docx]

Table S2

| **Prey Items** | **Water Canyon (n=12)** | **Ford Point (n=11)** | **Sandy Point (n=9)** | **Soledad (n=19)** | **China Camp (n=21)** | **SE Anchorage (n=10)** | **Bechers Bay (n=9)** | **Forneys Cove (n=11)** | **Coches Prietos (n=15)** | **Christy Beach (n=13)** |
| --- | --- | --- | --- | --- | --- | --- | --- | --- | --- | --- |
| **Beach** |  |  |  |  |  |  |  |  |  |  |
| **Crustacean** |  |  |  |  |  |  |  |  |  |  |
| *Megalorchestia spp.* (Amphipoda) | 17 |  | 22 | 58 | 24 | 30 | 22 | 73 | 13 | 85 |
| *Alloniscus perconvexus* (Isopoda) | 8 |  |  | 11 | 5 |  | 11 |  | 33 |  |
| *Tylos punctatus* (Isopoda) |  |  |  |  |  |  |  | 9 | 13 | 15 |
| *Emerita analoga* (Decapoda) |  |  |  |  |  | 10 |  |  |  |  |
| *Pleuroncodes planipes* (Decapoda) |  | 9 | 22 | 5 |  |  |  |  |  |  |
| Unidentified crab (Decapoda) |  |  |  | 5 |  | 20 |  | 9 | 13 |  |
| **Insect** |  |  |  |  |  |  |  |  |  |  |
| *Thinopinus pictus* (Coleoptera) | 17 |  |  | 37 | 14 |  | 11 | 27 | 20 | 85 |
| *Pontamalota opaca* (Coleoptera) |  |  |  |  |  |  |  | 9 |  |  |
| *Cafius spp.* (Coleoptera) |  |  |  | 16 |  |  |  |  |  |  |
| Histeridae (Coleoptera) |  |  |  | 5 |  |  |  |  |  |  |
| Unidentified (Coleoptera) |  |  |  |  | 5 |  |  |  | 13 |  |
| *Coelopa* pupa (Diptera) |  |  |  |  |  |  |  | 18 |  |  |
| Unidentified pupa (Diptera) |  |  |  |  |  |  |  | 9 | 7 |  |
| Unidentified larva (Diptera) |  |  |  |  |  |  |  | 18 |  |  |
| **Other beach** |  |  |  |  |  |  |  |  |  |  |
| *Strongylocentrotus purpuratus* (Echinoida) |  |  |  |  |  |  |  |  | 7 |  |
| **Terrestrial** |  |  |  |  |  |  |  |  |  |  |
| **Deer mouse (*Peromyscus maniculatus*)** |  |  |  |  |  |  |  |  |  |  |
| hair | 17 | 91 | 67 | 68 | 90 | 30 | 44 | 18 | 20 | 46 |
| bones | 8 | 82 | 44 | 53 | 86 | 30 | 11 | 18 |  | 23 |
| **Unidentified bird & other** |  |  |  |  |  |  |  |  |  |  |
| feathers (small) |  |  |  | 11 | 5 | 50 | 11 | 18 | 33 | 8 |
| bones |  |  |  | 11 | 5 | 30 | 11 | 9 | 7 | 8 |
| claw |  | 9 | 11 |  |  |  | 11 |  |  |  |
| Other (unidentified scales) |  |  |  |  | 5 |  |  |  |  |  |
| **Insect** |  |  |  |  |  |  |  |  |  |  |
| Jerusalem cricket (*Stenopelmatus* sp.) | 33 | 64 | 44 | 47 | 67 | 60 | 44 | 82 | 40 | 62 |
| Grasshopper (Orthoptera) | 25 | 18 | 44 | 21 | 14 | 20 | 33 | 9 | 7 | 31 |
| Cricket (Orthoptera) |  |  |  |  | 5 |  | 11 |  |  |  |
| Earwig (Dermaptera) |  | 18 |  | 11 | 14 |  | 11 |  | 13 | 54 |
| Terrestrial beetle (Tenebrionidae) |  | 18 |  | 5 | 14 |  | 33 |  | 27 | 15 |
| Other terrestrial insect |  |  | 11 | 5 |  |  | 22 |  |  | 8 |
| Terrestrial Isopod (*Porcellio* sp.) |  | 9 |  | 5 | 38 |  |  |  |  |  |
| **Plant** |  |  |  |  |  |  |  |  |  |  |
| Manzanita (*Arctostaphylos* sp.) | 100 |  |  |  |  | 60 | 22 |  | 60 |  |
| Australian saltbush (*Atriplex semibaccata*) |  | 45 |  | 53 | 71 | 20 | 56 | 55 |  | 31 |
| Ice plant (*Caprobrotus* sp.) | 8 |  | 22 | 26 | 29 |  | 11 |  | 46 | 46 |
| Summer holly (*Comarostaphylis diversifolia*) |  |  |  |  |  |  |  |  |  |  |
| Fruit/seeds (total) | 100 | 45 | 22 | 63 | 81 | 60 | 78 | 55 | 60 | 62 |
| Leaves/plant material | 33 | 9 | 11 | 11 | 14 | 20 | 22 | 27 | 7 | 31 |
| **Other terrestrial** |  |  |  |  |  |  |  |  |  |  |
| Snail |  |  | 11 | 5 |  |  |  |  |  |  |
| Sediment |  |  | 11 | 5 | 5 |  |  |  | 33 | 8 |
| **Anthropogenic** (fishing lures) |  |  |  |  | 5 | 10 |  |  |  | 8 |
