# Supplementary material for: Diet of a threatened endemic fox reveals variation in sandy beach resource use on California Channel Islands
Source: PLoS One. 2021 Oct 28;16(10):e0258919. doi: 10.1371/journal.pone.0258919 (PMC8553077; doi:10.1371/journal.pone.0258919)
Supplement: S3 Table — Results of Šidák post hoc pairwise comparison tests of invertebrate A) abundance and B) biomass between beaches on Santa Rosa and Santa Cruz Islands. SO—Soledad, SP—Sandy Point, SE—Southeast Anchorage, CC—China Camp, BB—Bechers Bay, WC—Water Canyon, FP—Ford Point, CB—Christy, FC—Forney’s Cove, CP—Coches Prietos. *Santa Cruz Island. Only values with significant differences (p < 0.05) shown). (DOCX) [file pone.0258919.s003.docx]

Table S3

| A. | SO | | SP | | SE | | CC | | BB | | WC | FP | | CB* | FC* | | CP* |  |
| --- | --- | --- | --- | --- | --- | --- | --- | --- | --- | --- | --- | --- | --- | --- | --- | --- | --- | --- |
| SO |  | | ns | | ns | | 0.001 | | 0.001 | | <0.001 | <0.001 | | ns | ns | | ns |  |
| SP |  | |  | | ns | | 0.02 | | 0.017 | | <0.002 | <0.001 | | ns | ns | | ns |  |
| SE |  | |  | |  | | ns | | ns | | 0.012 | <0.001 | | ns | <0.001 | | ns |  |
| CC |  | |  | |  | |  | | 0.001 | | 0.046 | <0.001 | | 0.001 | ns | | ns |  |
| BB |  | |  | |  | |  | |  | | ns | <0.001 | | 0.001 | ns | | ns |  |
| WC |  | |  | |  | |  | |  | |  | ns | | <0.001 | <0.001 | | <0.001 |  |
| FP |  | |  | |  | |  | |  | |  |  | | <0.001 | <0.001 | | ns |  |
| CB* |  | |  | |  | |  | |  | |  |  | |  | <0.001 | | ns |  |
| FC* |  | |  | |  | |  | |  | |  |  | |  |  | | ns |  |
| CP* |  | |  | |  | |  | |  | |  |  | |  |  | |  |  |
|  |  | |  | |  | |  | |  | |  |  | |  |  | |  |  |
| B. | SO | | SP | | SE | | CC | | BB | | WC | FP | | CH* | FC* | | CP* |  |
| SO |  | | ns | | ns | | ns | | ns | | <0.001 | <0.001 | | ns | ns | | ns |  |
| SP |  | |  | | ns | | ns | | ns | | <0.002 | <0.001 | | ns | ns | | ns |  |
| SE |  | |  | |  | | ns | | ns | | 0.012 | 0.001 | | ns | ns | | ns |  |
| CC |  | |  | |  | |  | | ns | | 0.016 | 0.004 | | ns | ns | | ns |  |
| BB |  | |  | |  | |  | |  | | ns | 0.017 | | ns | ns | | ns |  |
| WC |  | |  | |  | |  | |  | |  | ns | | <0.001 | 0.007 | | 0.002 |  |
| FP |  | |  | |  | |  | |  | |  |  | | <0.001 | 0.002 | | 0.001 |  |
| CB* |  | |  | |  | |  | |  | |  |  | |  | ns | | ns |  |
| FC* |  | |  | |  | |  | |  | |  |  | |  |  | | ns |  |
| CP* |  |  | |  | |  | |  | |  | | |  | | |  | | |
