# Supplementary material for: Diet of a threatened endemic fox reveals variation in sandy beach resource use on California Channel Islands
Source: PLoS One. 2021 Oct 28;16(10):e0258919. doi: 10.1371/journal.pone.0258919 (PMC8553077; doi:10.1371/journal.pone.0258919)
Supplement: S4 Table — Mean ± SE. *Santa Cruz Island. (DOCX) [file pone.0258919.s004.docx]

Table S4

| Site | Abundance (No. m^-1^ beach) | Biomass (g m^-1^ beach) |
| --- | --- | --- |
| Sandy Point | 4125 ± 1235 | 2300 ± 5912 |
| Soledad | 3132 ± 938 | 1680 ± 527 |
| China Camp | 2311 ± 1255 | 1066 ± 427 |
| Ford Point | 7754 ± 2236 | 1564 ± 458 |
| Bechers Bay | 12650 ± 4313 | 6398 ± 2140 |
| Water Canyon | 4329 ± 1385 | 3160 ± 1252 |
| SE Anchorage | 2682 ±769 | 1459 ± 337 |
| Forney Cove* | 3251 ± 1861 | 396 ± 123 |
| Christy Beach* | 18768 ± 10166 | 3617 ±1000 |
| Coches Prietos* | 862 ± 652 | 92 ± 36 |
