# Supplementary material for: Diet of a threatened endemic fox reveals variation in sandy beach resource use on California Channel Islands
Source: PLoS One. 2021 Oct 28;16(10):e0258919. doi: 10.1371/journal.pone.0258919 (PMC8553077; doi:10.1371/journal.pone.0258919)
Supplement: S5 Table — No fish were observed. Values in parentheses are number of fresh carcasses out of total, otherwise values represent old/desiccated carcasses. Blank cell = 0. Year of island fox scat collection (2018) in boldface. *Santa Cruz Island. (DOCX) [file pone.0258919.s005.docx]

| Table S5 |  | Live | | Carcass | | | | | | |
| --- | --- | --- | --- | --- | --- | --- | --- | --- | --- | --- |
|  |  | Pinnipeds | | Pinniped | | | Bird | | | Invertebrate |
| Beach site | Year | Elephant seal | Harbor seal | Elephant seal | Sea lion | Unk pinniped | Gull | Cormorant | Shearwater | Pelagic red crab |
| Bechers | 2016 |  |  |  |  |  |  |  |  |  |
|  | 2017 |  |  |  |  |  |  |  |  |  |
|  | **2018** |  |  |  |  |  |  |  |  |  |
| China Camp | 2016 | 300 |  | 1 |  |  |  |  |  |  |
|  | 2017 | 259 |  |  | 3 |  |  |  |  |  |
|  | **2018** | **63** |  |  |  | **3** |  |  |  |  |
| Ford Pt | 2016 | 9 | 6 |  |  | 1 |  |  |  |  |
|  | 2017 |  |  |  |  |  |  |  |  |  |
|  | **2018** |  |  |  |  |  |  |  |  |  |
| Sandy Pt | 2016 |  |  |  | 3 |  | 1 | 1 |  | 12-16 ind m^-1^ |
|  | 2017 |  |  |  |  |  |  |  |  |  |
|  | **2018** |  |  |  | **1** |  |  |  |  |  |
| Soledad | 2016 |  |  |  | 1 (1) |  |  | 1 |  | Common |
|  | 2017 |  |  |  |  |  |  |  |  |  |
|  | **2018** |  |  |  |  |  |  |  | **2** |  |
| Water Canyon | 2016 |  |  |  |  |  |  |  |  |  |
|  | 2017 |  |  |  | 1 |  |  |  |  |  |
|  | **2018** |  |  |  |  |  |  |  |  |  |
| Christy* | 2016 |  |  |  |  |  | 1 (1) |  |  |  |
|  | 2017 |  |  | 2 | 2 |  | 1 | 1 |  |  |
|  | **2018** |  |  |  | **1** |  |  |  |  |  |
| Coches* | 2016 |  |  |  |  |  |  |  |  |  |
|  | 2017 |  |  |  |  |  |  |  |  |  |
|  | **2018** |  |  |  | **1 (1)** |  |  |  |  |  |
| Forneys* | 2016 |  |  |  |  |  |  |  |  |  |
|  | 2017 |  |  |  |  |  |  |  |  |  |
|  | **2018** |  |  |  |  |  |  |  |  |  |
