# Supplementary material for: Diet of a threatened endemic fox reveals variation in sandy beach resource use on California Channel Islands
Source: PLoS One. 2021 Oct 28;16(10):e0258919. doi: 10.1371/journal.pone.0258919 (PMC8553077; doi:10.1371/journal.pone.0258919)
Supplement: S6 Table — Schamel et al., unpublished. (DOCX) [file pone.0258919.s006.docx]

Table S6

| Year | Dry Canyon | China Camp |
| --- | --- | --- |
| 2009 | 7 | 3 |
| 2010 | 5 | 5 |
| 2011 | 8 | 8 |
| 2012 | 16 | 9 |
| 2013 | 8 | 10 |
| 2014 | 11 | 7 |
| 2015 | 17 | 12 |
| 2016 | 18 | 8 |
| 2017 | 17 | 5 |
| 2018 | 21 | 6 |
| 2019 | 19 | 8 |
