# Supplementary material for: Diet of a threatened endemic fox reveals variation in sandy beach resource use on California Channel Islands
Source: PLoS One. 2021 Oct 28;16(10):e0258919. doi: 10.1371/journal.pone.0258919 (PMC8553077; doi:10.1371/journal.pone.0258919)
Supplement: S7 Table — CC = China Camp, SO = Soledad, AS = Arlington Springs. Segment 1 is the most proximal segment. n/d = no data. (DOCX) [file pone.0258919.s007.docx]

Table S7

| Site | Fox ID | Whisker Segment | δ^13^C | δ^15^N |
| --- | --- | --- | --- | --- |
| CC | 37503 | 1 | -19.0 | 11.2 |
| CC | 37503 | 2 | -18.4 | 10.8 |
| CC | 37503 | 3 | -18.6 | 10.8 |
| CC | 37503 | 4 | -19.0 | 9.9 |
| CC | 37503 | 5 | -19.5 | 11.0 |
| CC | 37503 | 6 | -19.3 | 10.9 |
| CC | 37503 | 7 | -19.8 | 10.9 |
| CC | 37503 | 8 | -20.2 | 11.1 |
| CC | 37503 | 9 | -19.9 | 11.4 |
| CC | 37503 | 10 | -20.2 | 11.3 |
| CC | 37503 | 11 | -20.5 | 10.8 |
| CC | 37547 | 1 | -20.1 | 11.9 |
| CC | 37547 | 2 | -19.0 | 11.3 |
| CC | 37547 | 3 | -18.6 | 10.8 |
| CC | 37547 | 4 | -18.3 | 11.3 |
| CC | 37547 | 5 | -18.0 | 11.3 |
| CC | 37547 | 6 | -18.0 | 10.6 |
| CC | 37547 | 7 | -18.1 | 10.8 |
| CC | 37547 | 8 | -18.5 | 10.6 |
| CC | 37547 | 9 | -19.2 | 10.7 |
| CC | 37547 | 10 | -19.0 | 10.7 |
| CC | 37547 | 11 | -19.0 | 10.7 |
| CC | 37547 | 12 | -20.1 | 10.9 |
| CC | 58012 | 1 | -20.0 | 12.2 |
| CC | 58012 | 2 | -20.3 | 12.3 |
| CC | 58012 | 3 | -20.2 | 11.9 |
| CC | 58012 | 4 | -19.5 | 12.0 |
| CC | 58012 | 5 | -18.1 | 12.7 |
| CC | 58012 | 6 | -18.3 | 12.5 |
| CC | 58012 | 7 | -19.1 | 12.0 |
| CC | 58012 | 8 | -19.3 | 12.3 |
| CC | 58012 | 9 | -20.1 | 12.4 |
| CC | 61904 | 1 | -19.5 | 12.6 |
| CC | 61904 | 2 | -19.2 | 12.2 |
| CC | 61904 | 3 | -19.4 | 12.4 |
| CC | 61904 | 4 | -19.7 | 12.4 |
| CC | 61904 | 5 | -19.5 | 12.2 |
| CC | 61904 | 6 | -19.3 | 12.3 |
| CC | 61904 | 7 | -18.7 | 12.0 |
| CC | 61904 | 8 | -17.4 | 12.4 |
| CC | 61904 | 9 | -17.4 | 12.4 |
| CC | 61904 | 10 | -18.8 | 12.1 |
| CC | 79756 | 1 | -19.7 | 11.3 |
| CC | 79756 | 2 | -19.2 | 12.1 |
| CC | 79756 | 3 | -19.5 | 11.6 |
| CC | 79756 | 4 | -18.9 | 11.8 |
| CC | 79756 | 5 | -18.9 | 11.2 |
| CC | 79756 | 6 | -19.2 | 11.7 |
| CC | 79756 | 7 | -18.9 | 11.8 |
| CC | 79756 | 8 | -18.5 | 11.5 |
| CC | 79756 | 9 | -17.9 | 11.2 |
| CC | 79756 | 10 | -18.1 | 12.3 |
| CC | 79756 | 11 | n/d | 11.5 |
| CC | A2B2C | 1 | -18.6 | 12.5 |
| CC | A2B2C | 2 | -20.5 | 10.9 |
| CC | A2B2C | 3 | -20.5 | 11.6 |
| CC | A2B2C | 4 | -20.5 | 11.3 |
| CC | A2B2C | 5 | -20.1 | 11.0 |
| CC | A2B2C | 6 | -19.9 | 11.0 |
| CC | A2B2C | 7 | -19.1 | 10.5 |
| CC | A2B2C | 8 | -18.6 | 11.6 |
| CC | A2B2C | 9 | -18.0 | 12.5 |
| CC | 23989 | 1 | -17.6 | 11.5 |
| CC | 23989 | 2 | -18.8 | 13.2 |
| CC | 23989 | 3 | -18.6 | 12.9 |
| CC | 23989 | 4 | -18.5 | 13.2 |
| CC | 23989 | 5 | -17.7 | 13.2 |
| CC | 23989 | 6 | -16.8 | 13.2 |
| CC | 23989 | 7 | -16.8 | 13.5 |
| CC | 23989 | 8 | -16.6 | 13.8 |
| CC | 23989 | 9 | -16.9 | 13.9 |
| CC | 23989 | 10 | -16.4 | 13.9 |
| CC | 80296 | 1 | -17.7 | 12.2 |
| CC | 80296 | 2 | -17.1 | 11.6 |
| CC | 80296 | 3 | -17.4 | 11.6 |
| CC | 80296 | 4 | -16.9 | 11.7 |
| CC | 80296 | 5 | -17.7 | 11.2 |
| CC | 80296 | 6 | -17.7 | 11.6 |
| CC | 80296 | 7 | -18.3 | 11.5 |
| CC | 80296 | 8 | -18.9 | 11.3 |
| CC | 80296 | 9 | -19.0 | 11.3 |
| CC | 80296 | 10 | -19.2 | 11.5 |
| CC | 80296 | 11 | -19.9 | 11.3 |
| SO | 80155 | 1 | -21.5 | 10.8 |
| SO | 80155 | 2 | -21.3 | 10.9 |
| SO | 80155 | 3 | -21.4 | 10.2 |
| SO | 80155 | 4 | -21.5 | 10.6 |
| SO | 80155 | 5 | -21.8 | 10.4 |
| SO | 80155 | 6 | -21.3 | 10.3 |
| SO | 80155 | 7 | -21.7 | 10.6 |
| SO | 80155 | 8 | -21.1 | 10.5 |
| SO | 80155 | 9 | -20.2 | 10.8 |
| SO | 80155 | 10 | -21.0 | 10.8 |
| SO | 80155 | 11 | n/d | n/d |
| SO | 80155 | 12 | -21.0 | 11.6 |
| SO | 80030 | 1 | -20.1 | 12.8 |
| SO | 80030 | 2 | -18.9 | 13.1 |
| SO | 80030 | 3 | -19.3 | 12.7 |
| SO | 80030 | 4 | n/d | n/d |
| SO | 80030 | 5 | -20.1 | 13.3 |
| SO | 80030 | 6 | -20.9 | 12.1 |
| SO | 80030 | 7 | -20.9 | 12.5 |
| SO | 80030 | 8 | -19.8 | 12.4 |
| SO | 80030 | 9 | -19.9 | 13.1 |
| SO | 80030 | 10 | -18.7 | 12.9 |
| SO | 80030 | 11 | -18.9 | 12.4 |
| SO | 80030 | 12 | nd | nd |
| SO | 80030 | 13 | -18.9 | 12.9 |
| SO | 80089 | 1 | -20.3 | 13.2 |
| SO | 80089 | 2 | -19.7 | 13.0 |
| SO | 80089 | 3 | -19.1 | 13.3 |
| SO | 80089 | 4 | nd | nd |
| SO | 80089 | 5 | -17.6 | 13.2 |
| SO | 80089 | 6 | -17.1 | 13.3 |
| SO | 80089 | 7 | -18.1 | 13.2 |
| SO | 80089 | 8 | -19.2 | 12.5 |
| SO | 80089 | 9 | -18.8 | 12.6 |
| SO | 80089 | 10 | -19.1 | 12.7 |
| SO | 80089 | 11 | -19.6 | 11.9 |
| SO | 26199 | 1 | -19.9 | 12.6 |
| SO | 26199 | 2 | -18.6 | 12.3 |
| SO | 26199 | 3 | -19.2 | 12.3 |
| SO | 26199 | 4 | -20.3 | 11.9 |
| SO | 26199 | 5 | -19.7 | 13.4 |
| SO | 26199 | 6 | -18.9 | 11.9 |
| SO | 26199 | 7 | -16.8 | 12.8 |
| SO | 26199 | 8 | -17.7 | 12.3 |
| SO | 26199 | 9 | -17.4 | 12.2 |
| SO | 26199 | 10 | -17.3 | 13.0 |
| SO | 26199 | 11 | -17.8 | 12.7 |
| SO | 26199 | 12 | nd | nd |
| SO | 79892 | 1 | -22.3 | 10.9 |
| SO | 79892 | 2 | -21.8 | 10.7 |
| SO | 79892 | 3 | -21.9 | 10.8 |
| SO | 79892 | 4 | -21.5 | 10.8 |
| SO | 79892 | 5 | -22.0 | 11.0 |
| SO | 79892 | 6 | -21.7 | 10.4 |
| SO | 79892 | 7 | -20.7 | 11.1 |
| SO | 79892 | 8 | -20.9 | 10.9 |
| SO | 79892 | 9 | -21.1 | 11.3 |
| SO | 79607 | 1 | -21.9 | 10.5 |
| SO | 79607 | 2 | -21.9 | 10.1 |
| SO | 79607 | 3 | -20.7 | 10.3 |
| SO | 79607 | 4 | -20.9 | 9.5 |
| SO | 79607 | 5 | -21.0 | 9.7 |
| SO | 79607 | 6 | -20.4 | 10.8 |
| SO | 79607 | 7 | -20.7 | 11.6 |
| SO | 79607 | 8 | -20.6 | 10.8 |
| SO | 79607 | 9 | -20.6 | 10.9 |
| SO | 79607 | 10 | -21.2 | 11.1 |
| SO | 79607 | 11 | -22.0 | 11.0 |
| SO | 79607 | 12 | -20.8 | 9.5 |
| SO | 79607 | 14 | -20.8 | 10.7 |
| SO | 79607 | 15 | -21.8 | 10.3 |
| SO | 70636 | 1 | -19.6 | 13.1 |
| SO | 70636 | 2 | -20.3 | 13.2 |
| SO | 70636 | 3 | -19.1 | 13.7 |
| SO | 70636 | 4 | -19.3 | 13.2 |
| SO | 70636 | 5 | -19.6 | 14.8 |
| SO | 70636 | 6 | -20.7 | 12.7 |
| SO | 70636 | 7 | -20.4 | 13.0 |
| SO | 70636 | 8 | -20.8 | 13.0 |
| SO | 70636 | 9 | -19.4 | 12.7 |
| SO | 70636 | 10 | -19.7 | 12.7 |
| SO | 70636 | 11 | -19.5 | 13.1 |
| SO | 70636 | 12 | -18.1 | 13.6 |
| SO | 70636 | 13 | -18.8 | 13.0 |
| SO | 1852 | 1 | -16.8 | 14.8 |
| SO | 1852 | 2 | -18.0 | 14.0 |
| SO | 1852 | 3 | -17.4 | 14.3 |
| SO | 1852 | 4 | -14.7 | 14.5 |
| SO | 1852 | 5 | -13.1 | 13.9 |
| SO | 1852 | 6 | -16.0 | 14.3 |
| SO | 1852 | 7 | -18.3 | 13.7 |
| SO | 1852 | 8 | -17.6 | 13.6 |
| SO | 1852 | 9 | -16.5 | 13.6 |
| SO | 1852 | 10 | -15.4 | 13.4 |
| SO | 1852 | 11 | -12.1 | 14.1 |
| SO | 1852 | 12 | -12.9 | 14.1 |
| SO | 1852 | 13 | -15.0 | 13.6 |
| SO | 1852 | 14 | -19.5 | 12.4 |
| SO | 1852 | 15 | -18.2 | 12.8 |
| SO | 79743 | 1 | -19.3 | 12.7 |
| SO | 79743 | 2 | -20.1 | 12.7 |
| SO | 79743 | 3 | -19.5 | 12.7 |
| SO | 79743 | 4 | -17.7 | 12.7 |
| SO | 79743 | 5 | -17.9 | 13.1 |
| SO | 79743 | 6 | -18.4 | 13.0 |
| SO | 79743 | 7 | -19.4 | 13.0 |
| SO | 79743 | 8 | -19.2 | 12.9 |
| SO | 79743 | 9 | -17.5 | 13.0 |
| SO | 79743 | 10 | -17.2 | 13.0 |
| SO | UNK | 1 | -19.0 | 12.8 |
| SO | UNK | 2 | -18.7 | 12.8 |
| SO | UNK | 3 | -19.7 | 12.7 |
| SO | UNK | 4 | -19.6 | 12.9 |
| SO | UNK | 5 | -19.3 | 12.9 |
| SO | UNK | 6 | -18.2 | 12.6 |
| SO | UNK | 7 | -18.1 | 13.0 |
| SO | UNK | 8 | -18.2 | 13.3 |
| SO | UNK | 9 | -18.2 | 12.7 |
| SO | UNK | 10 | -19.7 | 12.8 |
| AS | 79585 | 1 | -17.6 | 13.6 |
| AS | 79585 | 2 | -20.0 | 13.3 |
| AS | 79585 | 3 | -19.8 | 13.1 |
| AS | 79585 | 4 | -19.6 | 13.1 |
| AS | 79585 | 5 | -20.3 | 13.2 |
| AS | 79585 | 6 | -20.9 | 12.5 |
| AS | 79585 | 7 | -21.1 | 12.5 |
| AS | 79585 | 8 | -21.3 | 12.8 |
| AS | 79585 | 9 | -21.3 | 12.2 |
| AS | 79585 | 10 | -21.5 | 12.2 |
| AS | 79585 | 11 | -21.2 | 12.5 |
| AS | 79938 | 1 | -20.9 | 12.0 |
| AS | 79938 | 2 | -20.5 | 12.1 |
| AS | 79938 | 3 | -19.9 | 12.5 |
| AS | 79938 | 4 | -19.5 | 12.8 |
| AS | 79938 | 5 | -19.8 | 13.0 |
| AS | 79938 | 6 | -18.8 | 13.7 |
| AS | 79938 | 7 | -19.9 | 12.6 |
| AS | 79938 | 8 | -21.3 | 11.5 |
| AS | 79938 | 9 | -20.3 | 12.3 |
| AS | 79938 | 10 | -21.0 | 11.8 |
| AS | 79938 | 11 | -20.8 | 12.3 |
| AS | 79938 | 12 | -21.1 | 12.6 |
| AS | 91353 | 1 | -19.1 | 13.1 |
| AS | 91353 | 2 | -17.2 | 13.3 |
| AS | 91353 | 3 | -14.9 | 13.8 |
| AS | 91353 | 4 | -13.9 | 13.9 |
| AS | 91353 | 5 | -14.1 | 13.8 |
| AS | 91353 | 6 | -16.9 | 13.1 |
| AS | 91353 | 7 | -17.1 | 13.2 |
| AS | 91353 | 8 | -17.5 | 13.1 |
| AS | 91353 | 9 | -17.7 | 13.2 |
| AS | 91353 | 10 | -18.6 | 13.2 |
| AS | 91353 | 11 | -19.2 | 12.8 |
| AS | 80166 | 1 | -19.1 | 13.1 |
| AS | 80166 | 2 | -17.2 | 13.3 |
| AS | 80166 | 3 | -14.9 | 13.8 |
| AS | 80166 | 4 | -13.9 | 13.9 |
| AS | 80166 | 5 | -14.1 | 13.8 |
| AS | 80166 | 6 | -16.9 | 13.1 |
| AS | 80166 | 7 | -17.1 | 13.2 |
| AS | 80166 | 8 | -17.5 | 13.1 |
| AS | 80166 | 9 | -17.7 | 13.2 |
| AS | 80166 | 10 | -18.6 | 13.2 |
| AS | 80166 | 11 | -19.2 | 12.8 |
| AS | 80337 | 1 | nd | nd |
| AS | 80337 | 2 | -20.1 | 11.1 |
| AS | 80337 | 3 | -19.9 | 11.2 |
| AS | 80337 | 4 | -20.2 | 11.1 |
| AS | 80337 | 5 | -20.8 | 11.1 |
| AS | 80337 | 6 | -20.9 | 11.1 |
| AS | 80337 | 7 | -20.3 | 11.3 |
| AS | 80337 | 8 | -20.5 | 11.4 |
| AS | 80337 | 9 | -20.8 | 11.2 |
| AS | 80337 | 10 | -20.9 | 11.4 |
| AS | 80337 | 11 | -20.9 | 11.6 |
| AS | 80337 | 12 | nd | nd |
| AS | 80337 | 13 | -21.3 | 11.5 |
